# Unlocking the Phosphoric Acid Catalyzed Asymmetric Transfer Hydrogenation of 2-Alkenyl Quinolines for Efficient Flow Synthesis of Hancock Alkaloids

**DOI:** 10.1021/acs.orglett.5c00842

**Published:** 2025-03-21

**Authors:** Bence S. Nagy, Aitor Maestro, Miquel A. Pericàs, C. Oliver Kappe, Sándor B. Ötvös

**Affiliations:** † Institute of Chemistry, University of Graz, NAWI Graz, A-8010 Graz, Austria; ‡ Department of Organic Chemistry I, University of the Basque Country, UPV/EHU, Paseo de la Universidad 7, 01006 Vitoria-Gasteiz, Spain; § Universitat Rovira i Virgili, Departament de Química Física i Inorgànica, C/Marcel·lí Domingo 1, 43007 Tarragona, Spain; ∥ Center for Continuous Flow Synthesis and Processing (CC FLOW), Research Center Pharmaceutical Engineering GmbH (RCPE), A-8010 Graz, Austria

## Abstract

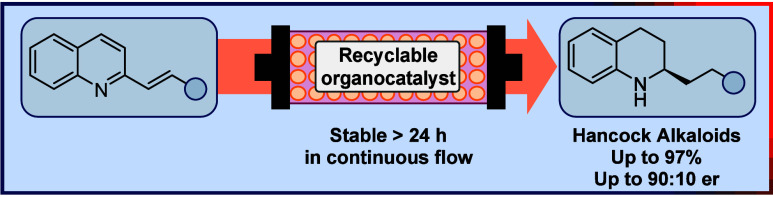

Chiral tetrahydroquinolines are present in several bioactive molecules, such as the Hancock alkaloids. Although the organocatalytic asymmetric transfer hydrogenation of 2-aryl quinolines has emerged as a safer alternative to using hydrogen gas, analogous reactions with 2-alkenyl quinolines remain unexplored. Here we present a protocol to synthesize key enantioenriched intermediates to the Hancock alkaloids, providing a constant outcome for, at least, a 24 h continuous flow operation.

Tetrahydroquinolines are biologically relevant molecules present in various natural products and active pharmaceutical ingredients (APIs). In particular, 2-alkyl tetrahydroquinolines can widely be found as key structural elements in biologically active molecules,^1,2^ such as the alkaloids from *Galipea officinalis* Hancock (Figure 1).^3^ Although extracts from the bark of *Galipea officinalis* (also known as angostura, cuspa, or galipea) were widely used in the south-American traditional medicine in the 19th century,^4^ the isolation and characterization of these alkaloids were not achieved until the late 1990s and early 2000s.^5,6^ In recent decades, the Hancock alkaloids have gained more attention as antituberculosis, anticancer, or antibacterial agents^7−9^ and have shown promising molecular docking potential that may render them useful against respiratory diseases such as COVID-19.^10^ As a consequence, several synthetic routes to efficiently access these alkaloids have been developed.^11^

**Figure 1:**
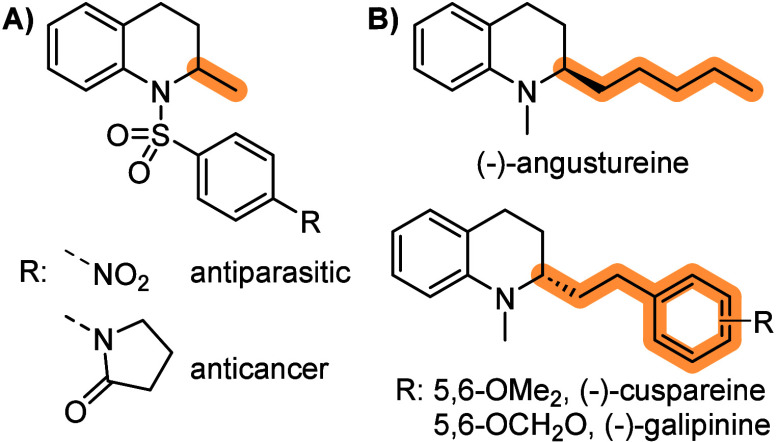
2-Alkyl tetrahydroquinoline derived APIs (A) and natural products from Galipea officinalis Hancock (B).

One of the most straightforward routes to access them is the hydrogenation of the corresponding quinolines or quinolones, using metal catalysts such as Pd, Pt, Ir, or Ru and hydrogen gas.^12−18^ However, the high cost and toxicity of precious metals, along with the risks derived from using hydrogen gas in large scales,^19,20^ necessitate other alternatives. In this context, transfer hydrogenation and its asymmetric variant, where a hydrogen precursor is used instead, represent a safer strategy.^21−24^ On the other hand, (chiral) Brønsted acids have demonstrated high effectiveness to catalyze this type of reaction using Hantzsch dihydropyridines and dihydrobenzothiazoles as the hydrogen source.^25−28^

In 2006, Rueping and co-workers^29^ reported the enantioselective transfer hydrogenation of quinolones catalyzed by chiral phosphoric acid organocatalysts, and since then, similar methodologies have been reported and applied to the synthesis of tetrahydroquinoline APIs.^25,26,30−33^ Due to its high conversion rates and low racemic background effects, this reaction has frequently been used in recent years for validating recyclable chiral phosphoric acid catalysts,^34−37^ with results often comparable to those obtained using the original homogeneous catalytic processes. However, the scope of the reaction is typically limited to 2-aryl quinolines along with scarce examples of substrate-specific transfer hydrogenations of 2-alkyl quinoline substrates.^38−41^ Despite the straightforward synthesis of 2-alkenyl quinolines,^42−44^ they have been largely overlooked for this type of reaction, highlighting the need for further development in asymmetric transfer hydrogenation processes.

In this paper, we report a novel methodology to access Hancock alkaloids via enantioselective transfer hydrogenation of 2-alkenyl quinolines using a heterogeneous chiral phosphoric acid organocatalyst (**PS-Anth**) in a packed-bed continuous flow reactor.

We first compared the performance of several chiral phosphoric acids toward the transfer hydrogenation of 2-alkyl, 2-alkenyl, and 2-pentynyl quinolines in the presence of Hantzsch ester **2** as the hydrogen source to access 2-pentyl tetrahydroquinoline **3a**, an advanced precursor of angustureine^3^ (Table 1). The initial experiments, carried out with 2-pentyl quinolone **1a**, revealed that catalysts bearing aromatic substituents (**I**–**IV**) afforded high yields in less than 1 h. However, catalyst **I** only afforded 65:35 er, while catalyst **II** resulted in an almost racemic product. On the other hand, the use of polyaromatic substituents on catalysts **III** and **IV** resulted in high yield and high enantioselectivity (er of 89:11 and 94:6, respectively). In contrast, highly sterically demanding catalyst **V** did not effectively catalyze the reaction, affording low yields even after 5 h. Next, we evaluated the use of alkenyl quinolone **1b**. Given that catalyst **I** has consistently induced high enantioselectivities with 2-aryl quinolines,^33,35,45−47^ we hypothesized that it would also be an effective choice for planar alkenyl groups. However, despite the clear increase in enantiocontrol with respect to quinolone **1a**, only moderate enantioselectivity was obtained. To our delight, catalysts **III** and **IV** afforded comparable yield and stereocontrol by slightly increasing the reaction time. Finally, we tested catalyst **IV** in the asymmetric transfer hydrogenation of 2-alkynyl quinoline **1c**. Interestingly, in this case, we did not observe the formation of tetrahydroquinoline **3a**.

**Table 1:**
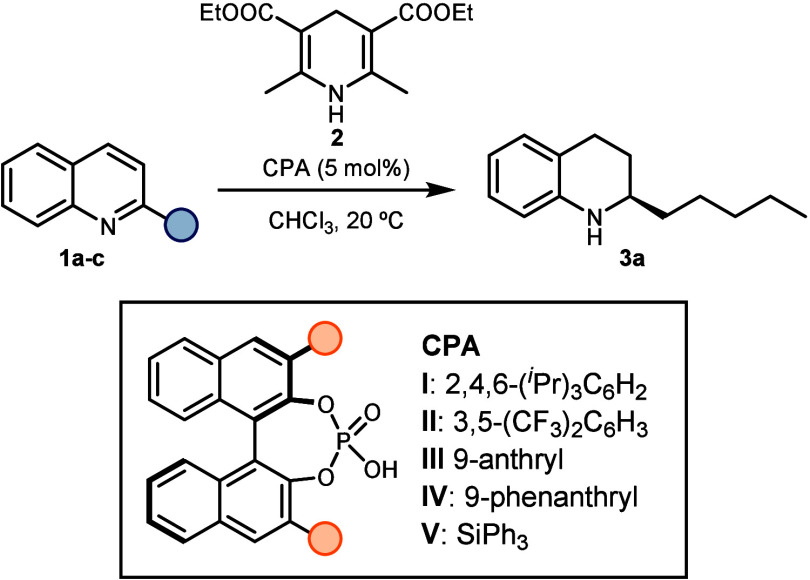

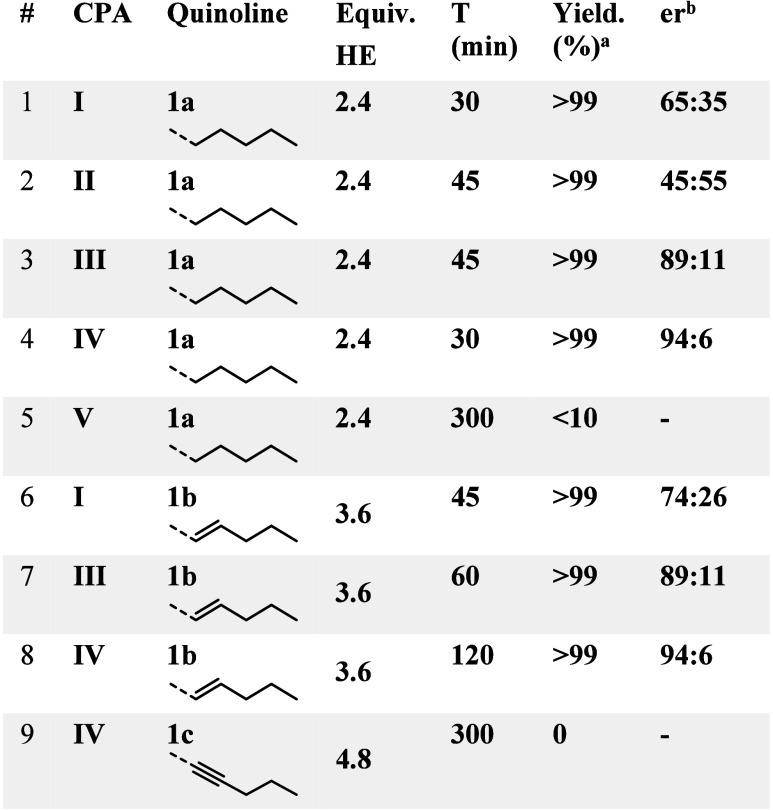
Testing Various Chiral Phosphoric Acids in the Asymmetric Transfer Hydrogenation of 2-Substituted Quinolines

a Determined by GC-FID Area %.

b Determined by chiral HPLC.

Recently, we have been active in the synthesis of highly recyclable chiral phosphoric acid organocatalysts as well as their applications for the continuous flow synthesis of various APIs and related intermediates.^31,48,49^ Additionally, we have devised a novel approach to access polystyrene-supported chiral phosphoric acids bearing 9-anthryl substituents as enantioselectivity directors (**PS-Anth**).^37^ Based on these backgrounds and also on the promising preliminary results obtained with some of the homogeneous catalysts in Table 1, we next wanted to explore the asymmetric transfer hydrogenation of 2-alkenyl quinolines under continuous flow conditions, thereby greatly facilitating scalability as well as environmental aspects of the protocol (Table 2). Although promising results were obtained with quinoline **1a**, 2-alkenylquinolines are more readily accessible than long-chain 2-alkylquinolines, which justifies their selection as substrates for flow experiments.

**Table 2:**
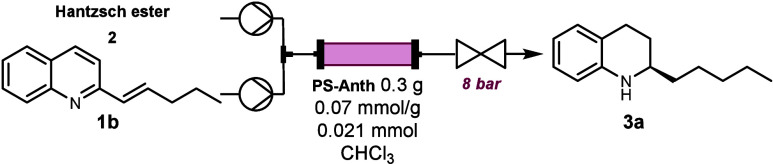
Optimization of the Asymmetric Transfer Hydrogenation of 2-Alkenyl Quinolines in Flow

#	P1, **2**/P2, **1b** (mL/min)	Flow rate (mL/min)	T (°C)	Yield (%)^a^	er^b^
1	0.25/0.25	0.50	20	39	82:18
2	0.25/0.25	0.50	50	77	86:14
3	0.20/0.20	0.40	50	86	86:14
4	0.15/0.15	0.30	50	94	86:14
5	0.10/0.10	0.20	50	>99	86:14

a Determined by GC-FID area %.

b Determined by chiral HPLC.

Our flow setup consisted of two independent streams for reagent **1b** (0.04 M, 1.0 equiv) and hydrogen source **2** (0.14 M, 3.5 equiv). The streams were combined right before entering a packed bed reactor containing 0.30 g of **PS-Anth**. The dissolution limit of Hantzsch ester **2** in CHCl_3_ was around 0.15 M; therefore, we decided to keep it at 0.14 M to avoid any incidental precipitation during the reaction. Considering that 3 equiv is required to fully reduce alkenyl quinolines, a 0.04 M solution of **1b** was used.

We did not explore any lower concentrations of reagents since it would strongly affect the final productivity. Initially, we tested the reaction at 0.5 mL/min overall flow rate and room temperature; however, low yields were obtained (Table 2, entry 1). Increasing the temperature to 50 °C allowed a good yield (77%) without losing enantiocontrol (Table 2, entry 2). Finally, the flow rate was reduced to 0.2 mL/min (i.e., 2 × 10 mL/min), achieving complete conversion to tetrahydroquinoline **3a**, with no reaction intermediate detected in the product as confirmed by GC-FID (Table 2, entries 3–5).

Therefore, the optimal setup required pumping **1b** and **2** each at 0.1 mL/min through a packed bed reactor with a calculated internal volume of 2.3 mL, resulting in an overall flow rate of 0.2 mL/min and an estimated residence time of 12 min (Scheme 1). To demonstrate the robustness of the setup, a 24 h continuous flow preparative run was carried out for the synthesis of angustureine intermediate **3a** using alkenyl quinoline **1b** as the precursor. During the initial 6 h of the experiment, the crude was collected into separate samples for 1 h each. Then, from 6 to 24 h, the reactor output was collected separately every 3 h. All fractions were analyzed independently by off-line GC-FID and chiral HPLC, obtaining almost quantitative yield (97%, 1.136 g) and 85:15 er in each sample, corresponding to a turnover number (TON) of 266. In contrast to other reports where chiral phosphoric acids suffer from deactivation after several hours and a reactivation protocol is needed,^36,50^ we observed stable yields and selectivities without detectable loss of catalytic activity during the process. Remarkably, due to the C(sp^3^)–C(sp^3^) binding of the catalyst to the polymer support, no catalyst leaching was observed. This is in agreement with previous data for analogous PS-TRIP and closely related catalysts.^35,49,51^ Unfortunately, **3a** was obtained as a liquid, and crystallization procedures to further increase the enantiomeric ratios were unsuccessful. Next, we extended the methodology to the synthesis of Hancock alkaloid precursors **3b** and **3c**. In these cases, the reactions were run continuously for 12 h, obtaining 0.719 g (84%) of tetrahydroquinoline **3b** (74:26 er) and 0.713 g (88%) of **3c** (90:10 er), respectively. This represents a TON of 115 and 121, for **3b** and **3c**, respectively. It should be noted that the sequential synthesis protocol was simple and effective with no observed catalyst deactivation over time and required only a 10 min washing cycle of the catalyst bed with CHCl_3_ between each run to avoid cross-contamination. Therefore, the accumulated TON combining all the preparative runs is above 500 with a total operation time of 48 h.

**Scheme 1:**
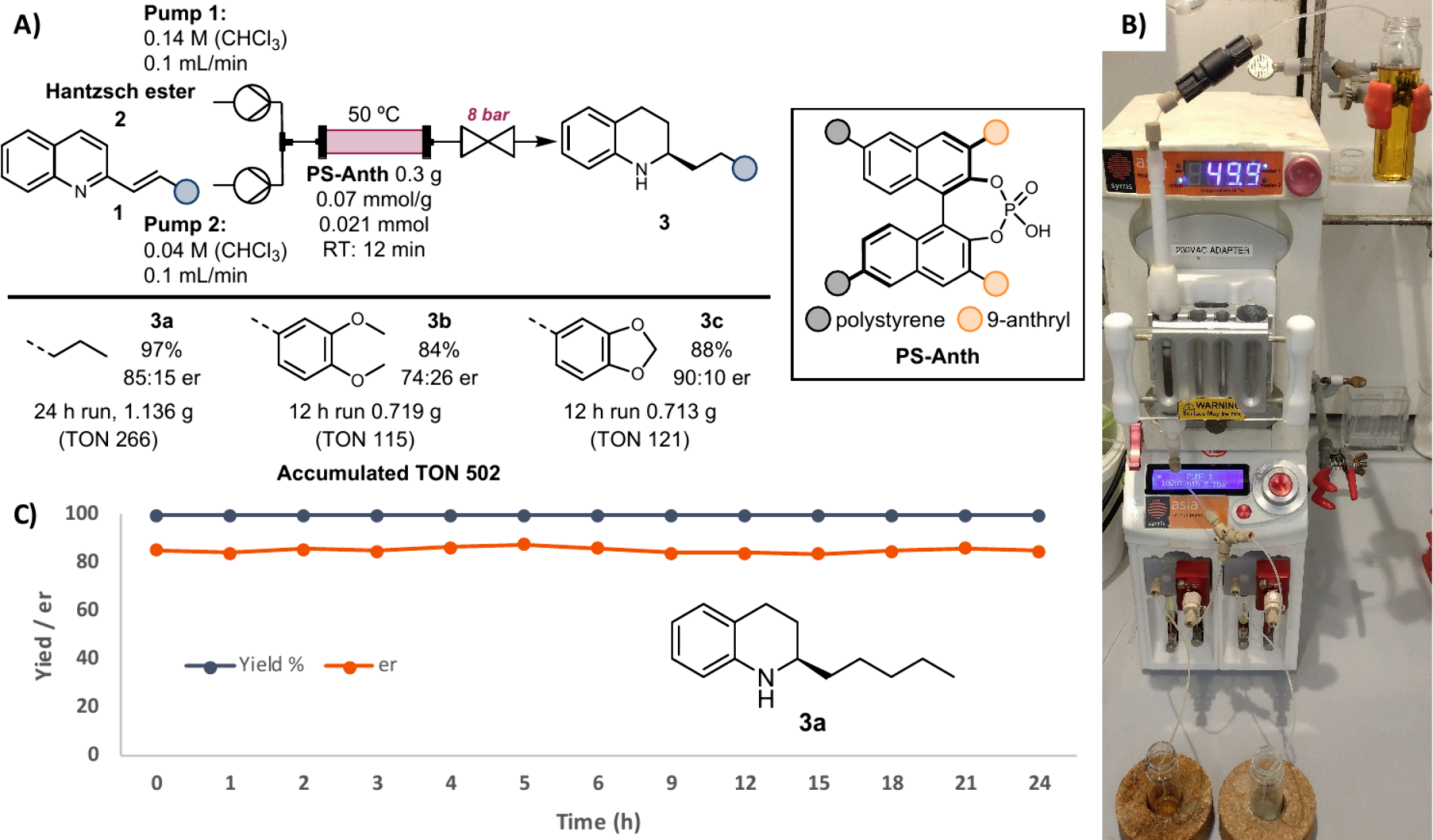
A) Schematic Representation of the Optimal Flow Setup and Reaction Scope, B) Photograph of the Setup during the Preparative Run, and C) Yield and er for the 24 h Preparative Run (**3a**) Isolated yields are shown. Yield (blue) was determined by GC-FID area %. er (orange) was determined by chiral HPLC area %; the major enantiomer is shown.

The most plausible reaction mechanism for the transfer hydrogenation of quinolines consists of an initial quinolinium salt formation (Scheme 2).^52^ Then, the catalyst mediates the asymmetric transfer hydrogenation to the quinolinium ion by activating the Hantzsch dihydropyridine through hydrogen bonding between the N–H group of the Hantzsch ester and the phosphoryl group. Subsequently, an alkene isomerization should occur, leading to a 1,4-dihydroquinoline. The enamine–imine equilibrium would allow for the second transfer hydrogenation. An analogous alkene isomerization/enamine–imine equilibrium might be involved in the hydrogenation of the 2-alkenyl moiety. However, during the optimization studies for the synthesis of Hancock alkaloid intermediates **3**, we were unable to detect any of the intermediates. We hypothesized that the highly conjugated systems in these compounds may stabilize the 2-alkenyl tetrahydroquinoline, making the final alkene isomerization more challenging and potentially preventing identification of the reaction intermediate. To clarify this, we performed a control experiment by modifying the reaction conditions for the synthesis of **3c**, using only 2.5 equiv of the Hantzsch ester. This led to a mixture of **3c** and intermediate **4** which was confirmed by NMR and GC-MS (Figures S2–S5). Further addition of Hantzsch ester to this **3c** + **4** mixture in the presence of the chiral catalyst did not increase the conversion to **3c**, meaning that alkene isomerization occurs only during the transfer hydrogenation process.

**Scheme 2:**
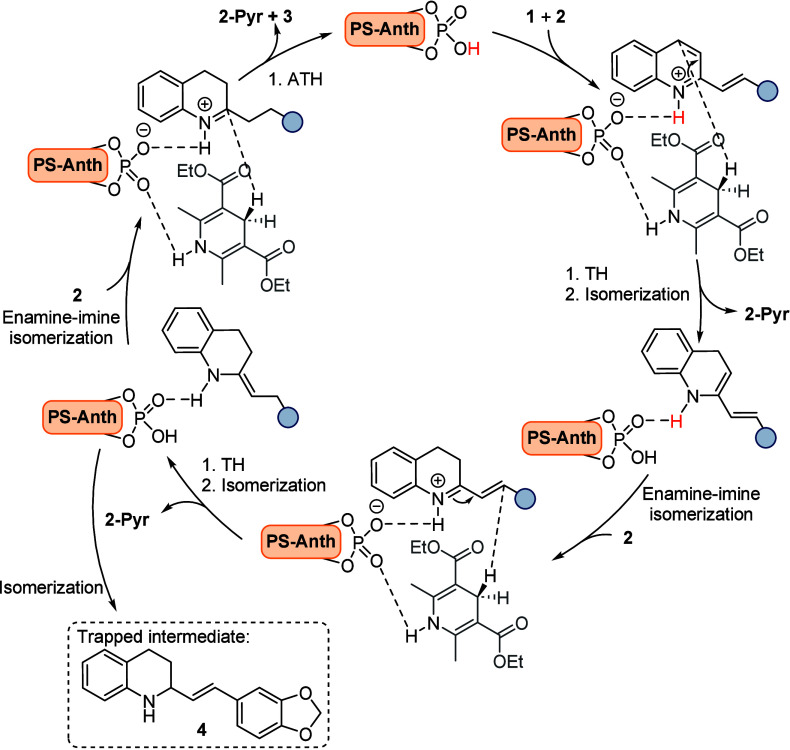
Proposed Mechanism for the Asymmetric Transfer Hydrogenation of 2-Alkenyl Quinolines

In summary, we have developed an unprecedented asymmetric transfer hydrogenation of readily available 2-alkenyl quinolines and successfully applied it to the synthesis of biologically active Hancock alkaloid intermediates **3**. The reaction has been optimized using both batch and continuous flow techniques. The flow setup using a highly recyclable **PS-Anth** catalyst showed high robustness for up to 48 h operation time, resulting in an overall catalyst loading of merely 0.2 mol % and an accumulated TON of >500. Additionally, a mechanistic proposal for the asymmetric transfer hydrogenation of 2-alkenyl quinolines was developed, supported by the identification of a trapped reaction intermediate (**4**).

## Data Availability

The data underlying this study are available in the published article and its Supporting Information.

## Supporting Information Available

General procedures and characterization data (PDF)
