# Supplementary material for: Unlocking the Phosphoric Acid Catalyzed Asymmetric Transfer Hydrogenation of 2-Alkenyl Quinolines for Efficient Flow Synthesis of Hancock Alkaloids
Source: Org Lett. 2025 Mar 21;27(13):3414–9. doi: 10.1021/acs.orglett.5c00842 (PMC11976859; doi:10.1021/acs.orglett.5c00842)
Supplement: Supplementary file 1 — ol5c00842_si_001.pdf [file ol5c00842_si_001.pdf]

Unlocking the phosphoric acid catalyzed asymmetric transfer hydrogenation of 2-alkenyl quinolines for efficient flow synthesis of Hancock alkaloids

Bence S. Nagy,[a] Aitor Maestro,*[a,b] Miquel A. Pericàs,[c] C. Oliver Kappe[a,d] and Sándor B. Ötvös*[a,d]

[a] Institute of Chemistry, University of Graz, NAWI Graz, A-8010 Graz, Austria; [b] Department of Organic Chemistry I, University of the Basque Country, UPV/EHU, Paseo de la Universidad 7, 01006 Vitoria-Gasteiz, Spain; [c] Universitat Rovira i Virgili, Departament de Química Física i Inorgànica, C/Marcel·lí Domingo 1, 43007 Tarragona, Spain; [d] Center for Continuous Flow Synthesis and Processing (CC FLOW), Research Center Pharmaceutical Engineering GmbH (RCPE), A-8010 Graz, Austria.

E-mail: aitor.maestro@ehu.eus; sandor.oetvoes@uni-graz.at

General Experimental Information....2
Synthesis and characterization of **PS-Anth**....3
Reaction optimization....9
Mechanistic investigations....10
Continuous flow synthesis and characterization of **3**....13
Chiral HPLC chromatograms of **3**....17
NMR spectra of BINOL derivatives **B-F**....20
NMR spectra of **3**....26
References....29

General Experimental Information

Solvents and chemicals were obtained from typical commercial vendors and were used as received, without any further purification. 2-substituted quinolines **1** were synthesized according to literature protocols.[1–4]

When required, column chromatographic purification was performed by using a Biotage Isolera automated flash chromatography system with cartridges packed with KP-SIL, 60 Å (32–63 µm particle size). Analytical thin-layer chromatography (TLC) was carried out using Merck silica gel 60 GF254 plates. Compounds were visualized by means of UV or by using KMnO4.

^{1}H, and ^{13}C-NMR spectra were recorded on a Bruker Avance III 300 MHz instrument at room temperature, in $CDCl_3$ as a solvent, at 300 MHz and 75 MHz, respectively. Chemical shifts (δ) are reported in ppm relative to the residual solvent peak ($CDCl_3$, ^{1}H: 7.26 ppm; ^{13}C: 77.16 ppm). Coupling constants are reported in Hertz. Multiplicity is reported with the usual abbreviations. Structural assignments were made with additional information from gCOSY, gHSQC, and gHMBC experiments.

GC analysis was performed on a Shimadzu GC FID 230 with a flame ionization detector (FID), using an RTX-5MS Cap. column (30 m × 0.25 mm ID × 0.25 µm) and helium as carrier gas (40 cm/sec-1 linear velocity). The injector temperature was set to 280 °C. After 1 min at 50 °C, the temperature was increased by 25 °C/min to 300 °C and kept constant at 300 °C for 4 min. FID was used for detection, and the detector gases used for flame ionization were hydrogen and synthetic air (5.0 quality).

GC-MS was performed using a Shimadzu GCMS-QP2010 SE, using a Rtx-5MS column (30 m × 0.25 mm × 0.25 µm) and helium as carrier gas with a linear velocity of 40 cm/sec. The injector temperature was set to 280 °C. After 1 min at 50 °C, the oven temperature was increased by 25 °C/min to 300 °C and then kept at 300 °C for 3 min. The mass detector was a quadrupole with pre-rods and electron impact ionization. The following settings were used in the detector: ion source temperature 200 °C, interface temperature 310 °C, solvent cut time 2 min 30 sec, acquisition mode scan, mass range m/z = 50 till m/z = 400.

Chiral HPLC analysis was performed on a Shimadzu HPLC system (DGU-403 degassing unit, CTO-40S column oven, CBM20 system controller, SPD-40 UV-VIS detector, LC-20AT pumps).

The absolute configuration was determined by comparison of chiral HPLC data with literature reports for compound **3a**, and the absolute configurations of other compounds were assigned by analogy.[S5]

High-resolution mass spectra were recorded in either negative or positive mode on an Agilent 6230 TOF LC/MS (G6230B) by flow injections on an Agilent 1260 Infinity Series HPLC (HiP Degasser G4225A, Binary Pump G1312B, ALS Autosampler G1329B, TCC Column thermostat G1316A, DAD Detector G4212B).

Equipment for the continuous flow reactions was assembled using commercially available components. Liquid streams were pumped by using Syrris® Asia syringe pumps. Reactor coils were made by using perfluoroalkoxy alkane (PFA) tubings (1/16" OD, 0.80 mm ID or 1/8" OD, 1.58 mm ID). Details of reaction setups as well as general procedures can be found in the following sections.

Infrared spectra (FTIR) of **PS-Anth** was taken in a Bruker Alpha spectrometer with an ATR unit.

Synthesis and characterization of **PS-Anth**[6]

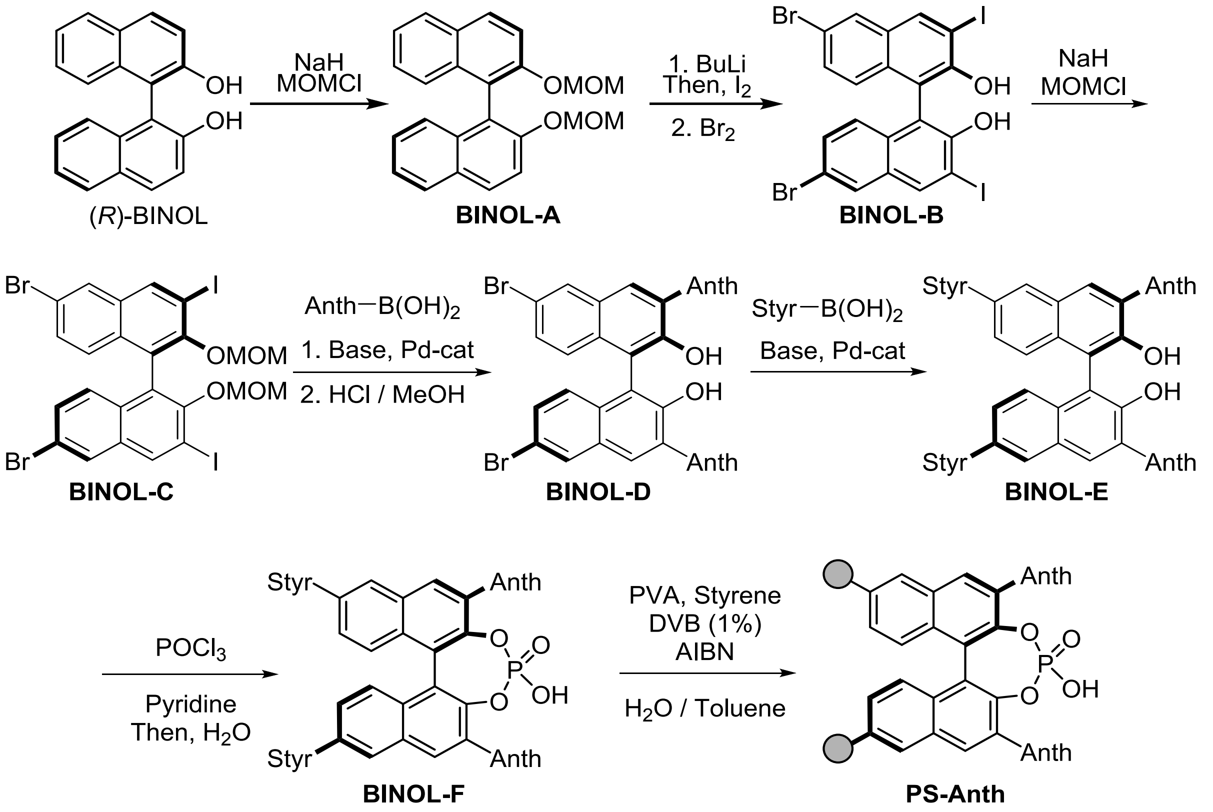

A suspension of NaH[1] (60 % dispersion in mineral oil, 5.00 g, 125.0 mmol, 2.5 equiv.) in anhydrous THF (50 mL) was cooled to 0°C in an ice bath and a solution of BINOL (14.32 g, 50.0 mmol, 1.0 equiv.) in THF (100 mL) was added dropwise over 10 min.[2] The reaction was stirred at room temperature for 1 h before the addition of MOMCl (9.50 mL, 125 mmol, 2.5 equiv.). The reaction mixture was stirred for 4 h at room temperature and quenched with water (30 mL). The organic layer was then separated and the aqueous phase was extracted with Et_2O (3 x 80 mL). The combined organic extracts were washed with brine (100 mL) and dried over Na_2SO_4, filtered, and concentrated under reduced pressure to afford the MOM-protected **BINOL-A** as an amorphous white solid (18.72 g, quant.).

To a solution of **BINOL-A** (18.72 g, 50 mmol, 1.0 equiv.) and TMEDA (18.74 mL, 125 mmol, 2.5 equiv.) in anhydrous THF (200 mL), n-BuLi (2.5M solution in hexanes, 48.00 mL, 120 mmol, 2.4 equiv.) was added dropwise at -80 °C under nitrogen atmosphere (EtOAc / Liq N_2 bath). The resulting solution was warmed up to 0 °C and stirred for 30 min. After cooling back to -80 °C, a solution of iodine (35.53 g, 140 mmol, 2.8 equiv.) in anhydrous THF was added dropwise and the mixture was slowly warmed up to room temperature and stirred overnight. The resulting mixture was opened to air and bromine (6.40 mL, 125 mmol, 2.5 equiv.) was added dropwise at 0 °C and the reaction was vigorously stirred at room temperature for 4 h. The excess of iodine and bromine was quenched with a saturated aqueous solution of Na_2SO_3 (100 mL). The organic layer was then separated and the aqueous phase was extracted with Et2O (3 x 40 mL). The combined organic extracts were washed with brine (50 mL) and dried over Na_2SO_4, filtered, and concentrated under vacuum to afford product **BINOL-B** as a yellow solid, which was purified by column chromatography on silica gel (hexanes / EtOAc) as an amorphous yellow solid (29.23 g, 84% over two steps).

---

[1] NaH was washed with hexanes prior to use to remove mineral oils.

[2] Caution! Strong H_2 evolution.

A suspension of NaH[3] (60 % dispersion in mineral oil, 2.00 g, 50.0 mmol, 2.5 equiv.) in anhydrous THF (50 mL) was cooled to 0°C in an ice bath and a solution of **BINOL-B** (13.92 g, 20.0 mmol, 1.0 equiv.) in THF (50 mL) was added dropwise over 10 min[4]. The reaction was stirred at room temperature for 1 h before the addition of MOMCl (3.80 mL, 50 mmol, 2.5 equiv.). The reaction mixture was stirred for 4 h at room temperature and quenched with water (10 mL). The organic layer was then separated and the aqueous phase was extracted with Et2O (3 x 40 mL). The combined organic extracts were washed with brine (50 mL) and dried over Na_2SO_4, filtered, and concentrated under reduced pressure to afford the product **BINOL-C** as an amorphous yellow solid (15.67 g, quant.).

Two necked round bottom flask equipped with a magnetic stirrer and a reflux condenser was charged **BINOL-C** (1.57 g, 2.0 mmol, 1.0 equiv.), Cs_2CO_3 (1.95 g, 6.0 mmol, 3.0 equiv.), $Pd(PPh_3)_4$ (115.6 mg, 0.1 mmol, 0.05 equiv.) and the 9-anthryl boronic acid (1.07 g, 4.8 mmol, 2.5 equiv.). The flask was then purged with Ar in a Schlenk line. Then, degassed dimethoxyethane (9 mL) and water (3 mL) were added through a septum and the reaction was stirred at 85 °C for 16 h (heating block). After cooling down to room temperature and washing with saturated $NaHCO_3$ (3 x 10 mL), MeOH (30 mL) and aqueous HCl (37%, 3 mL) were added, and the reaction was heated at 50 °C overnight. Then, the reaction mixture was cooled down, diluted in CH_2Cl_2, and the organic phases were washed with saturated $NaHCO_3$ (2 x 30 mL) and brine (30 mL), dried over Na_2SO_4, filtered and concentrated under vacuum to afford the crude 3,3´-diaryl **BINOL-D**, which was purified by chromatography on silica gel (hexanes / CH_2Cl_2), affording 1.18 g (74%) of the product as an amorphous pale yellow solid.

Two necked round bottom flask equipped with a magnetic stirrer and a reflux condenser was charged **BINOL-D** (0.80 g, 1.0 mmol, 1.0 equiv.), Cs_2CO_3 (0.98 g, 3.0 mmol, 3.0 equiv.), Pd(PPh3)4 (57.8 mg, 0.05 mmol, 0.05 equiv.) and the 4-styryl boronic acid (384.7 mg, 2.6 mmol, 3.5 equiv.). The flask was then purged with Ar in a Schlenk line. Then, degassed toluene (10 mL) and water (5 mL) were added through a septum and the reaction was stirred at 110 °C for 16 h (heating block). After cooling down to room temperature, the reaction mixture was filtered off through a short pad of celite and washed with CH_2Cl_2 (2 x 30 mL). The resulting biphasic mixture was washed with saturated $NaHCO_3$ (30 mL) and brine (30 mL), and the organic phase was dried over Na_2SO_4, filtered, and concentrated under vacuum to afford the crude **BINOL-E**, which was purified by chromatography on silica gel (hexanes / CH_2Cl_2), affording 767.2 mg (91%) of the product as an amorphous pale yellow solid.

BINOL derivative **BINOL-E** (843.0 mg, 1.0 mmol, 1.0 equiv.) was dissolved in anhydrous pyridine (5.0 mL) and $POCl_3$ (279.6 µL, 3.0 mmol, 3.0 equiv.) were added dropwise. Then, the reaction was heated at 95 °C for 8 h (heating block). The reaction was cold down to room temperature and H_2O (5.0 mL) was added dropwise. Then, the reaction mixture was heated at 95 °C for another 10 h (heating block). After completing the reaction, it was cold down to room temperature and acidified with 6 M HCl (40 mL). The reaction product was extracted from CH_2Cl_2 (50 mL) and the organic phase was sequentially washed with 6 M HCl (2x 30 mL) to fully acidify the phosphoric acid. The organic phase was then dried over $MgSO_4$ and filtered to afford 886.9 mg (98%) of the product as an amorphous pale yellow solid.

[3] NaH was washed with hexanes prior to use to remove mineral oils.

[4] Caution! Strong H_2 evolution.

(*R*)-6,6'-dibromo-3,3'-diiodo-[1,1'-binaphthalene]-2,2'-diol (BINOL-B)

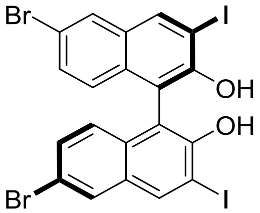

Purified by column chromatography on silica gel (hexanes / EtOAc). Spectroscopic data matches the literature.[S6,7]

^{1}H NMR (300 MHz, $CDCl_3$) δ 8.40 (s, 2H), 7.94 (d, *J* = 2.0 Hz, 2H), 7.37 (dd, *J* = 9.0, 2.0 Hz, 2H), 6.90 (d, *J* = 9.0 Hz, 2H), 5.44 (s, 2H).

^{13}C {^{1}H} NMR (75 MHz, $CDCl_3$) δ 150.5, 139.4, 131.9, 131.6, 131.4, 129.3, 126.3, 118.7, 112.9, 88.6.

HRMS (TOF-) m/z: Calcd for $C_{20}H_9Br_2I_2O_2$ $[M-H]^-$: 692.7064; Found: 692.7056.

(*R*)-6,6'-dibromo-3,3'-diiodo-2,2'-bis(methoxymethoxy)-1,1'-binaphthalene (BINOL-C)

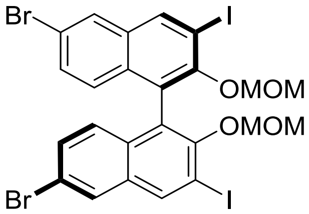

No additional purification was required. Spectroscopic data matches the literature.[S6]

^{1}H NMR (300 MHz, $CDCl_3$) δ 8.45 (s, 2H), 7.94 (d, *J* = 2.0 Hz, 2H), 7.37 (dd, *J* = 9.0, 2.0 Hz, 2H), 7.01 (d, *J* = 9.0 Hz, 2H), 4.80 (d, *J* = 5.9 Hz, 2H), 4.74 (d, *J* = 5.9 Hz, 2H), 2.57 (s, 6H).

^{13}C {^{1}H} NMR (75 MHz, $CDCl_3$) δ 153.0, 139.3, 133.1, 132.3, 130.7, 128.8, 128.3, 126.1, 120.1, 99.8, 94.1, 56.6, 27.0.

HRMS (TOF+) m/z: Calcd for $C_{26}H_{25}Br_2I_2O_5S$ $[M+DMSO+H]^+$: 860.7873; Found: 860.7848.

(*R*)- 6,6'-dibromo-3,3'-di(anthracen -9-yl)-[1,1'-binaphthalene]-2,2'-diol (BINOL-D)

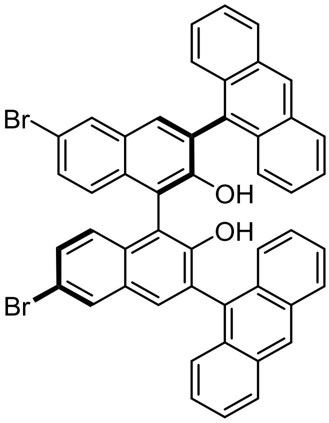

Purified by chromatography on silica gel (hexanes / CH_2Cl_2). Spectroscopic data matches the literature.[S6]

^{1}H NMR (300 MHz, $CDCl_3$) δ 8.60 (s, 2H), 8.14 – 8.03 (m, 6H), 7.92 (s, 2H), 7.82 – 7.76 (m, 2H), 7.64 – 7.40 (m, 12H), 7.30 – 7.22 (m, 2H), 5.02 (s, 2H).

^{13}C {^{1}H} NMR (75 MHz, $CDCl_3$) δ 151.2, 132.6, 132.1, 131.6, 131.6, 131.0, 130.8, 130.8, 130.5, 130.5, 129.6, 128.9, 128.8, 128.5, 128.4, 126.7 (m), 125.9, 125.6 (m), 125.6, 118.2, 114.1.

HRMS (TOF-) m/z: Calcd for $C_{48}H_{27}Br_2O_2$ $[M-H]^-$: 793.0383; Found: 793.0378.

(*R*)-3,3'-di(anthracen-9-yl)-6,6'-bis(4-vinylphenyl)-[1,1'-binaphthalene]-2,2'-diol (BINOL-E)

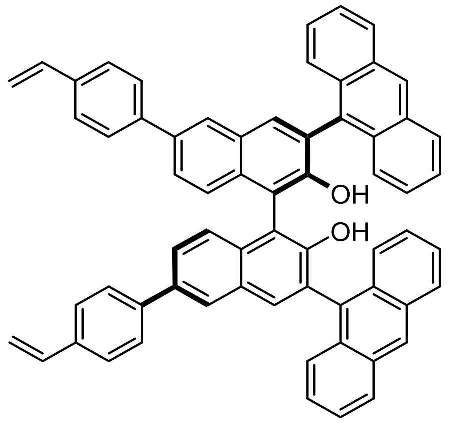

Purified by chromatography on silica gel (hexanes / CH_2Cl_2). Spectroscopic data matches the literature.[S6]

^{1}H NMR (300 MHz, $CDCl_3$) δ 8.63 (s, 2H), 8.25 – 8.03 (m, 8H), 8.02 – 7.93 (m, 2H), 7.85 – 7.71 (m, 8H), 7.62 – 7.41 (m, 12H), 7.35 – 7.29 (m, 2H), 6.81 (dd, *J* = 17.6, 11.0 Hz, 2H), 5.85 (d, *J* = 17.6 Hz, 2H), 5.32 (d, *J* = 11.0 Hz, 2H), 5.18 (s, 2H).

^{13}C {^{1}H} NMR (75 MHz, $CDCl_3$) δ 151.3, 140.4, 136.8, 136.7, 136.5, 133.4, 133.3, 131.7, 131.6, 131.0, 130.9, 130.6, 129.7, 128.9, 128.7, 128.1, 127.8, 127.5, 127.1, 126.9, 126.5, 126.3, 126.2, 125.6, 125.6, 114.1, 113.8.

HRMS (TOF+) m/z: Calcd for $C_{64}H_{43}O_2$ $[M+H]^+$: 843.3258; Found: 843.3260.

(*R*)-2,6-di(anthracen-9-yl)-4-hydroxy-9,14-bis(4-vinylphenyl)dinaphtho[2,1-d:1',2'-f][1,3,2]dioxaphosphepine 4-oxide (BINOL-F)

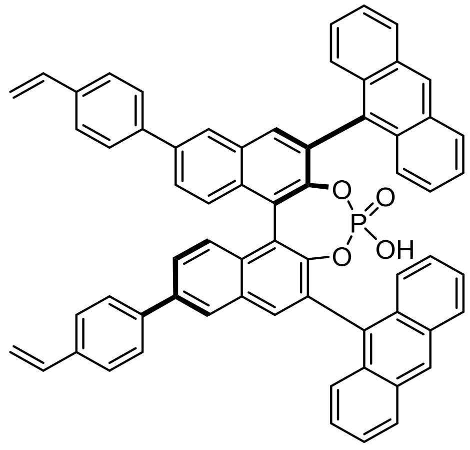

No additional purification was required. Spectroscopic data matches the literature.[S6]

^{1}H NMR (300 MHz, , DMSO) δ 8.65 (s, 2H), 8.57 – 8.35 (m, 2H), 8.19 – 8.05 (m, 6H), 7.93 – 7.83 (m, 6H), 7.65 – 7.28 (m, 18H), 6.80 (dd, *J* = 17.6, 11.0 Hz, 2H), 5.91 (d, *J* = 17.7 Hz, 2H), 5.30 (d, *J* = 11.2 Hz, 2H).

* The H from the phosphoric acid could not be observed.

^{13}C {^{1}H} NMR (75 MHz, DMSO) δ 149.7, 139.0, 136.7 – 135.6 (m), 133.8 – 124.4 (m), 122.5, 118.8, 114.5.

^{31}P NMR (121 MHz, DMSO) δ 2.66.

HRMS (TOF+) m/z: Calcd for $C_{64}H_{42}O_4P$ $[M+H]^+$: 905.2815; Found: 905.2832.

Method for the copolymerization of BINOL-F with styrene and divinylbencene

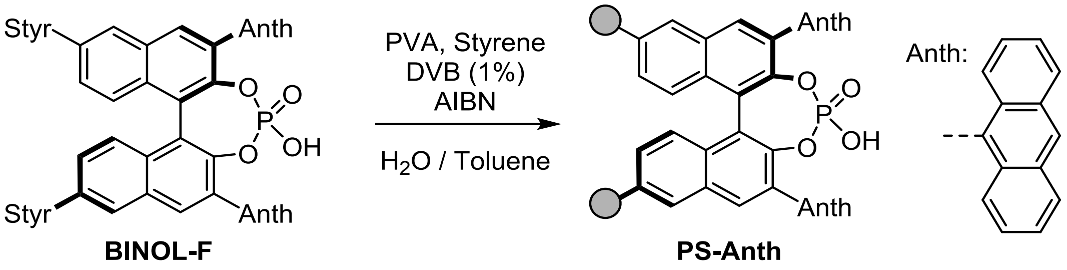

A round bottom flask was charged with a suspension of polyvinyl alcohol (PVA) (65 mg, 0.64 µmol, 0.001 equiv.) in 60 mL of deionized and degassed water at room temperature. After stirring at rt for 0.5 h, the solution was heated at 90 °C (heating block) until PVA was dissolved (1.5-2 h) and then cooled down to room temperature.

On the other hand, the reaction mixture was prepared in a different flask. First, 1,4-divinylbenzene (DVB 85% purity, 88 µL, 0.5 mmol, 0.8 equiv.) and styrene (2.66 mL, 23.1 mmol, 35.0 equiv.) were filtered through a short pad of silica to remove the stabilizers, and the filter was further washed with 3.0 mL of toluene. Then, **BINOL-F** (600 mg, 0.66 mmol, 1.0 equiv.) and AIBN (36 mg, 0.22 mmol, 0.33 equiv.) were dissolved in the DVB/Styrene/Toluene mixture and the resulting solution was degassed by bubbling Argon for 10 minutes.

The pre-made reaction mixture was added to the aqueous solution of PVA at room temperature. Additional 3.0 mL of degassed toluene was used to wash the flask containing the reaction mixture. Then, the reaction was stirred at 80 °C for 2 days (heating block). Then, the aqueous solution was decanted and the resin was washed with hot water (50 °C, 3x 100 mL), followed by MeOH (3x 50 mL) and CH_2Cl_2 (3x 50 mL). The resulting catalyst was dried overnight in a vacuum oven at 40 °C to afford 1.86 g of **PS-Anth** as brown beads.

The catalyst loading of the resin was calculated based on the P elemental analysis by using the following formula:

$$f\left(\frac{mmol}{g}\right) = \frac{\%P\ x\ 1000}{number\ of\ P\ atoms\ x\ MW(P) x\ 100}$$

Elem. Anal. P: 0.21%.

f = 0.07 mmol/g

FTIR

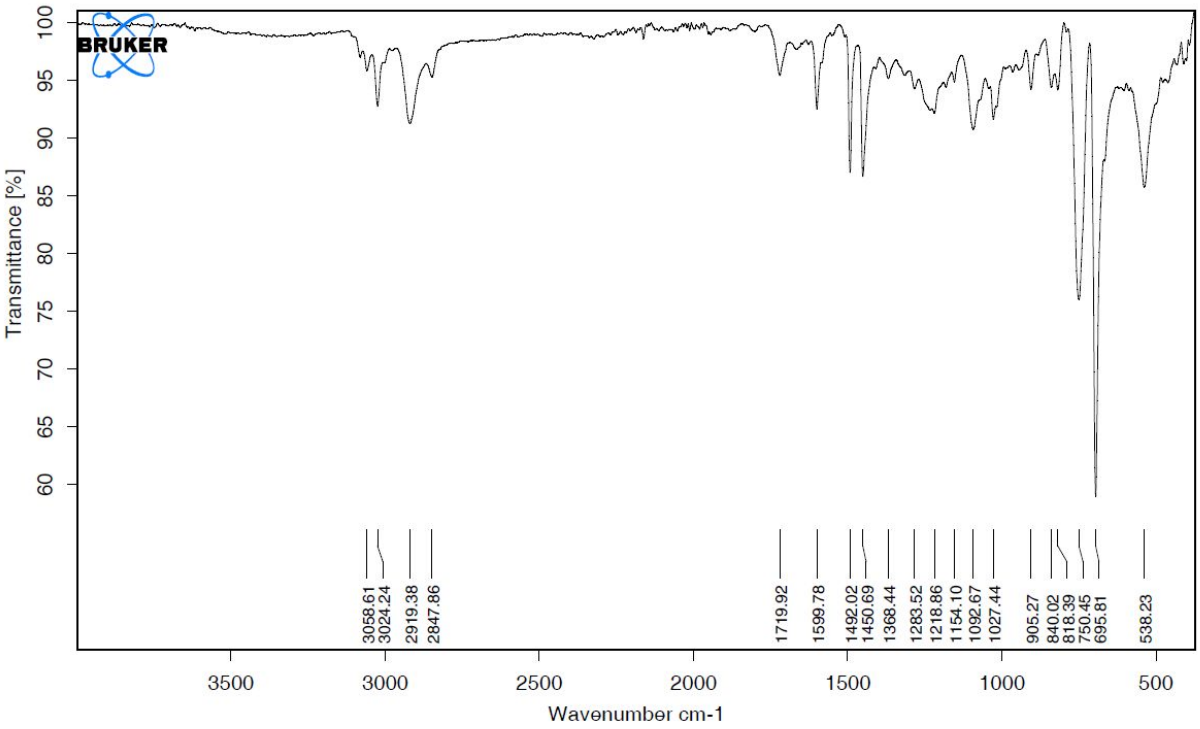

Figure S1. *FTIR of **PS-Anth** catalyst.*

Reaction optimization

Table S1. Batch synthesis of chiral tetrahydroquinoline **3a** *via* homogeneous CPA-catalyzed enantioselective transfer hydrogenation of quinolines **1a-c**.

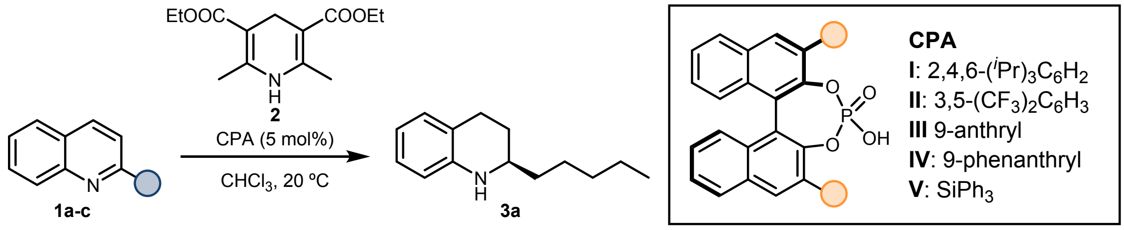

#	Catalyst	Quinoline	Equiv. HE	T (min)	Yield. (%)[a]	er[b]
1	**I**	**1a**	2.4	30	>99	65:35
2	**II**	**1a**	2.4	45	>99	45:55
3	**III**	**1a**	2.4	45	>99	89:11
4	**IV**	**1a**	2.4	30	>99	94:6
5	**V**	**1a**	2.4	300	0	-
6	**I**	**1b**	3.6	45	>99	74:26
7	**III**	**1b**	3.6	60	>99	89:11
8	**IV**	**1b**	3.6	120	>99	94:6
9	**IV**	**1c**	4.8	300	0	-

a) Determined by GC-FID Area %.
b) Determined by chiral HPLC.

To a solution of **1** (0.1 mmol, 1.0 equiv.) in $CHCl_3$ (1.0 mL) was added the corresponding chiral phosphoric acid (0.01 mmol, 0.1 equiv.) and Hantzsch ester **2** (0.24-0.48 mmol, 2.4-4.8 equiv.) at 20 °C. The reaction was stirred at 300 rpm for 0.5-5.0 h and monitored by GC-FID area %. Reaction products were identified by GC-MS.

Note: The racemic products were prepared using TFA (5 mol%) as the catalyst.

Mechanistic investigations

The reaction intermediate **4** was identified by following the general procedure for the reaction optimization using only 2.5 equivalents of the Hantzsch ester **2** and quinoline **1d**. The reaction mixture was purified by chromatography in silica gel (hexanes / EtOAc) to obain a mixture of **3c** and **4** (slightly contaminated with **2-Pyr** byproduct). Both, the pure **3c** and the **3c**/**4** mixture where characterized by ^{1}H-NMR (Figure S2) and COSY (Figures S3-4). The new peaks at 6.0 and 6.5 ppm confirm the presence of an alkene in the structure, while the signal at 4.0 ppm corresponds to the H in position 2 of the tetrahydroquinoline. Moreover, the mixture was also analyzed by GC-MS, confirming the presence of 2 molecules with m/z matching **3c** and **4** (Figure S5). Other potential isomers of **4** were discarded as those would not have any H in position 2, and would only present a single H for the alkene moiety.

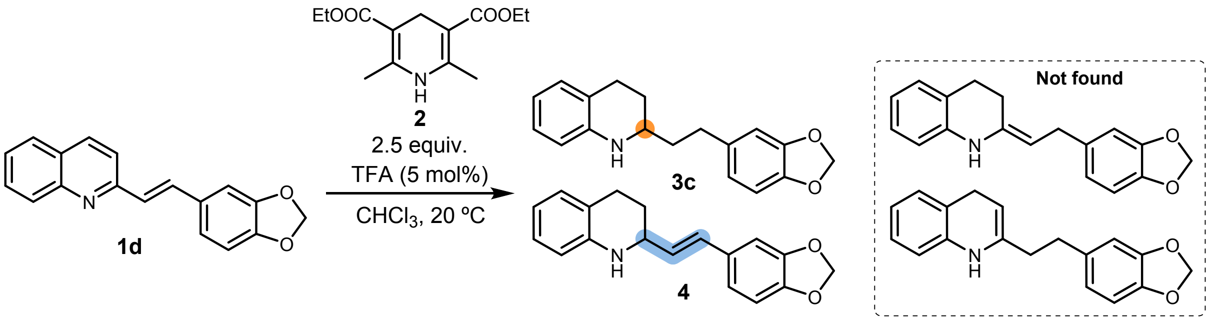

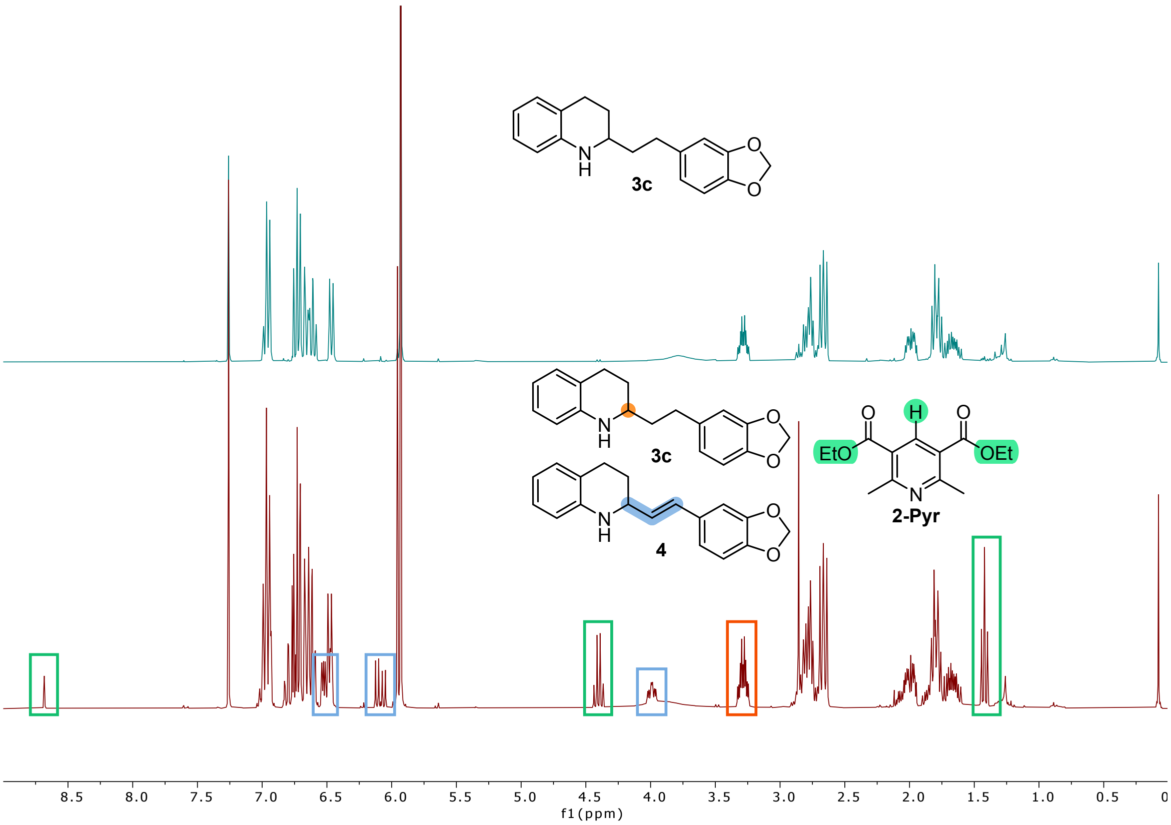

***Figure S2.** ^{1}H-NMR comparison of pure **3c** and **4** trapping experiment (mixture of **3c**, **4** and **2-Pyr**).*

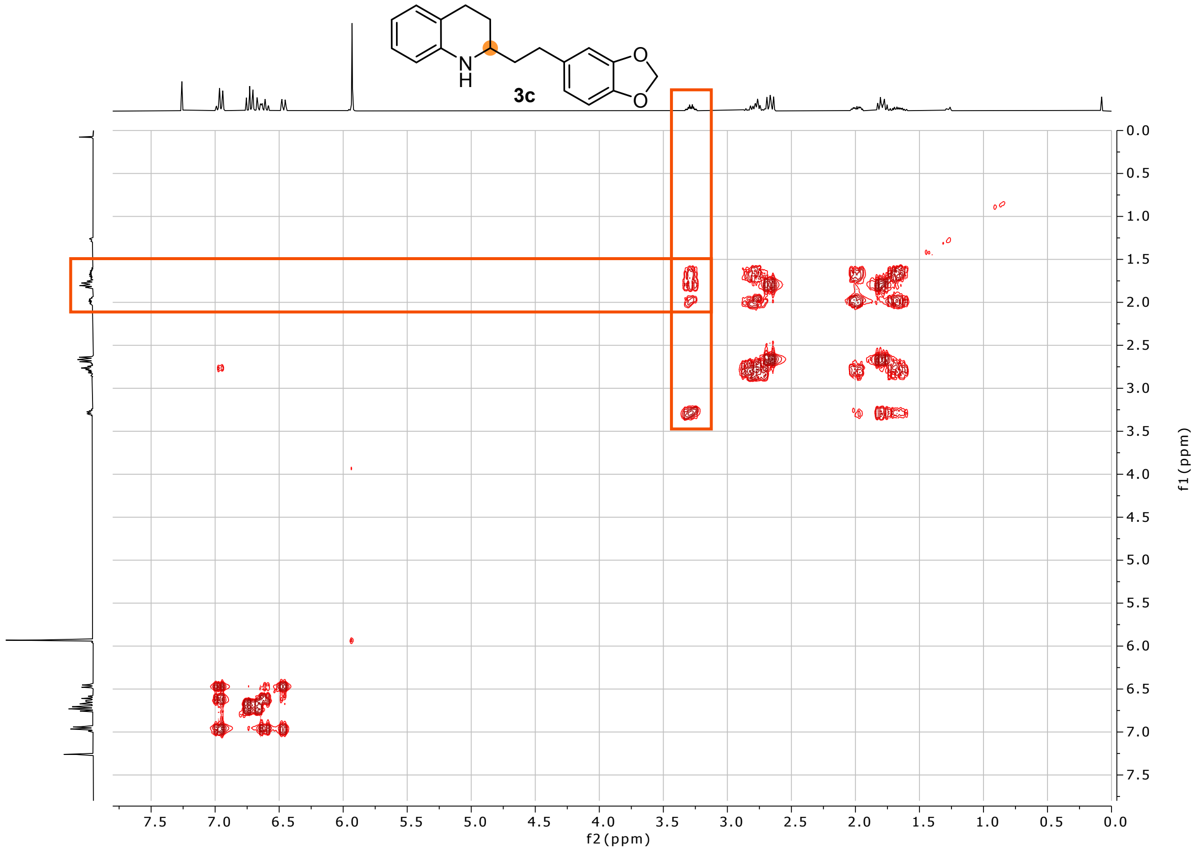

Figure S3. *COSY NMR of pure **3c**.*

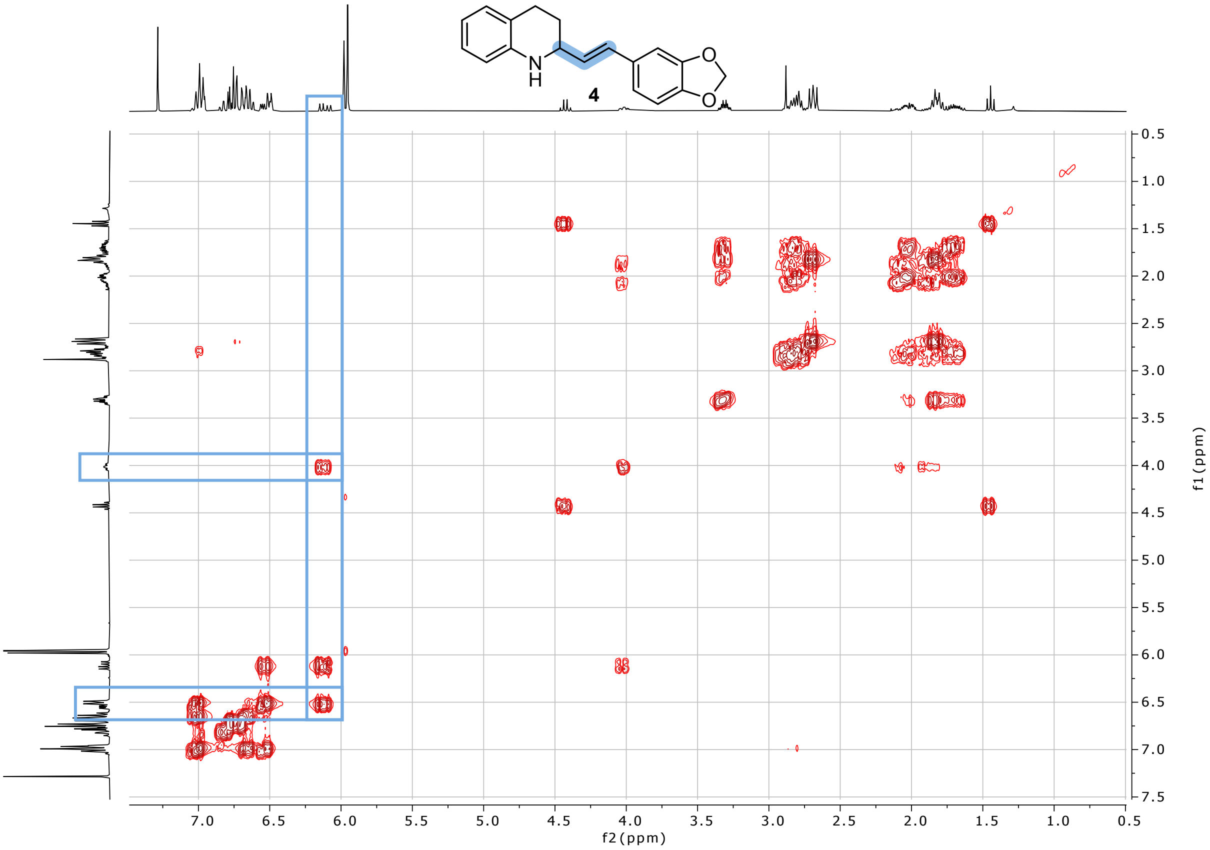

Figure S4. *COSY NMR of **4** trapping experiment (mixture of **3c**, **4** and **2-Pyr**).*

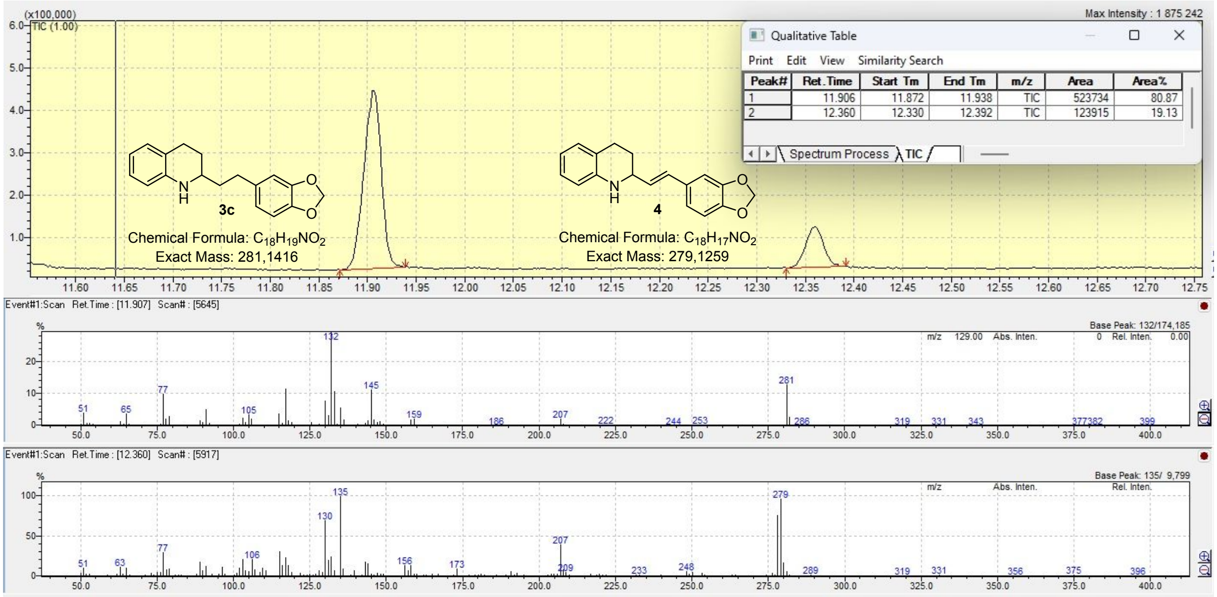

Figure S5. *GC-MS of **4** trapping experiment (mixture of **3c**, **4** is shown).*

Continuous flow synthesis and characterization of **3**

Optimization

Table S2. *Optimization of the asymmetric transfer hydrogenation of 2-alkenyl quinoline **1b** in flow.*

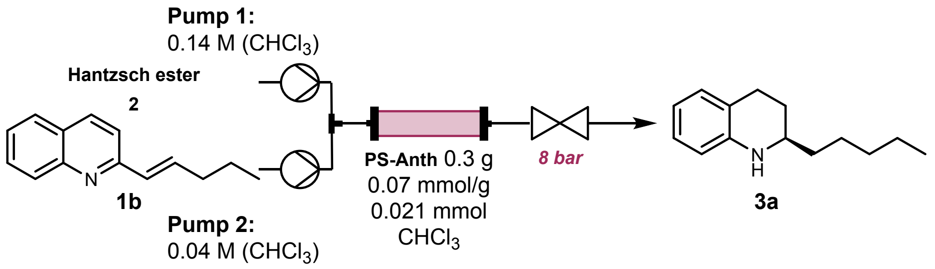

#	P1 (mL/min)	P2 (mL/min)	Flow rate (mL/min)	Equiv. HE	Conc. Quin. (M)	Conc. HE (M)	T (°C)	Yield (%)[a]	er[b]
**1**	0.25	0.25	0.50	3.5	0.02	0.07	20	39	82:18
**2**	0.25	0.25	0.50	3.5	0.02	0.07	50	77	86:14
**3**	0.20	0.20	0.40	3.5	0.02	0.07	50	86	86:14
**4**	0.15	0.15	0.30	3.5	0.02	0.07	50	94	86:14
**5**	0.10	0.10	0.20	3.5	0.02	0.07	50	>99	86:14

a) Determined by GC-FID Area %.
b) Determined by chiral HPLC.

Preparative run

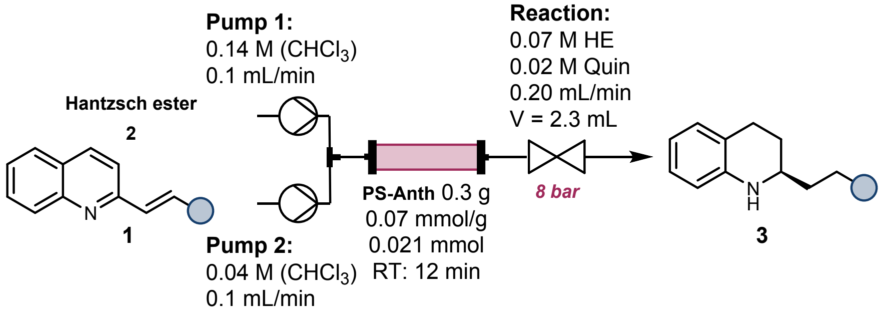

The packed bed reactor was filled with 0.3 g of **PS-Anth** (0.07 mmol/g, 0.021 mmol, dried overnight at 40°C in a vacuum oven). Then, the reaction was swollen with $CHCl_3$ and 1.2 g of glass beads were added on top. The adjustable end of the Omnifit® glass column (10 mm ID) was then closed and adjusted as required. Both ends were closed with a frit (See Figure S6).[5] Over all the project, the packed bed reactor was stored swollen in $CHCl_3$. Before each reaction, the set up was stabilized by pumping $CHCl_3$ at 0.5 mL/min for 5-10 min and the catalyst bed was pre-heated to 50 °C (packed-bed reactor heater). The stock solutions (in $CHCl_3$) of 2-alkenyl quinoline **1** (0.04 M, 0.10 mL/min, 1.0 equiv.) and **Hantzsch ester 2** (0.14 M, 0.10 mL/min, 3.5 equiv.) were pumped independently and pre-mixed right before entering the Omnifit column containing the **PS-Anth** catalyst by using a Syrris® Asia syringe pump (0.20 mL/min overall flow rate). The pressure was stable around 8.0 bar over the preparative runs. Between runs, the quinoline stream was washed by pumping $CHCl_3$ at 0.5 mL/min for 5 min.

[5] The use of glass beads was needed to avoid the catalyst particles to obstruct the frit.

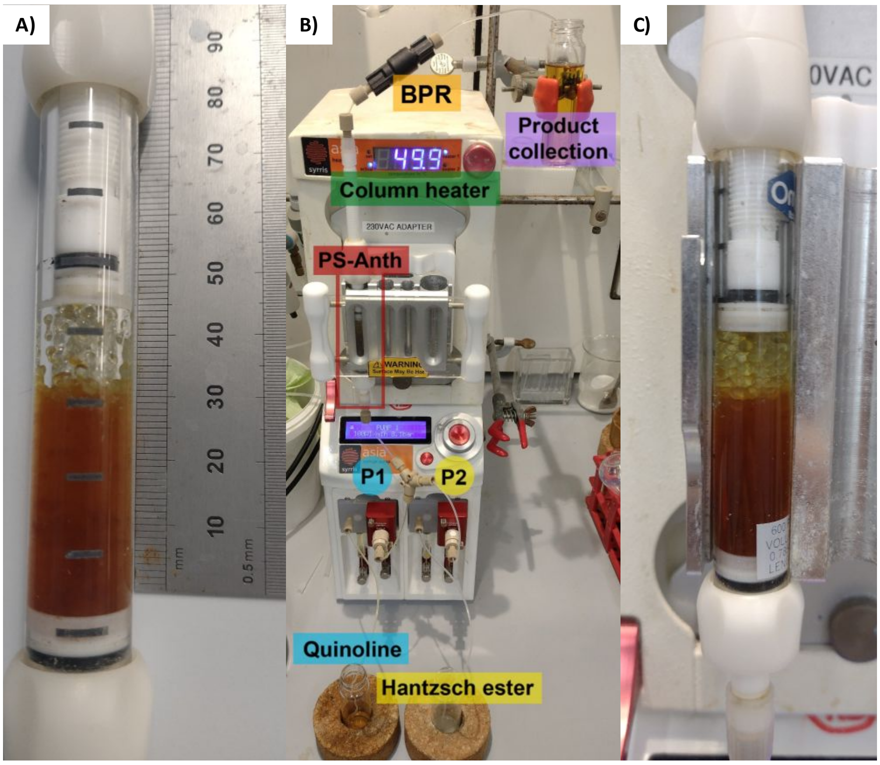

Figure S6. A) *Freshly prepared catalyst bed.* ***B)*** *Optimal flow set up.* ***C)*** *Catalyst bed during the preparative run of* ***3a****.*

The reactor volume was calculated using the packed bed reactor volume. It was calculated according to the indications from the vendor using the catalyst height.

$$\text{Bed volume (mL)} = 0.7854 \times \text{bed height (cm)} = 0.7854 \times 3.0 = 2.3562\ mL$$

Catalyst metrics for the preparative runs (3a-c)

Turnover number (TON) and space time yield (STY) and resin productivity were calculated for the each compound the literature formulas.[S8,9]

(*R*)-2-pentyl-1,2,3,4-tetrahydroquinoline (3a)

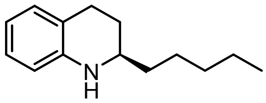

The general procedure was followed, collecting the reaction product for 24h in steady state and purified by column chromatography on silica gel (hexanes / EtOAc) to afford 1.136 g (97%) of the product as a brown liquidThe reported data match the literature. [S5,10]

^{1}H NMR (300 MHz, $CDCl_3$) δ 7.00 – 6.91 (m, 2H), 6.61 (td, *J* = 7.4, 1.1 Hz, 1H), 6.48 (d, *J* = 8.3 Hz, 1H), 3.29 – 3.14 (m, 2H), 2.91 – 2.63 (m, 2H), 2.07 – 1.88 (m, 1H), 1.66 – 1.31 (m, 9H), 0.91 (t, *J* = 6.8 Hz, 3H).

^{13}C {^{1}H} NMR (75 MHz, $CDCl_3$) δ 144.8, 129.4, 126.8, 121.6, 117.1, 114.3, 51.8, 36.8, 32.1, 28.2, 26.6, 25.5, 22.8, 14.2.

HRMS (TOF+) m/z: Calcd. for $C_{14}H_{22}N$ $[M+H]^+$: 204.1747; Found: 204.1750.

HPLC (chiral): Chiralpak-OJ-H, n-heptane/i-PrOH 95:5, 0.5 mL/min, 40 °C, detection at 254 nm.

Residence time: 9.6 min (minor), 10.2 (major). 85:15 er.

Lit. HPLC (chiral): Chiralpak-OJ-H, Hexanes/i-PrOH 95:5, 0.5 mL/min, temperature not specified, detection at 254 nm.

Residence time: 17.64 min (major), 19.22 (minor). *S*-isomer.[S5]

$$\text{TON} = \frac{mmoles\ limiting\ reactant}{mmoles\ catalyst} \times yield = \frac{5.76}{0.021} \times 0.97 = 266.1$$

$$\text{STY} = \frac{mass\ of\ product}{volume\ of\ reactor\ \times reaction\ time} = \frac{1.136\ e^{-3} kg}{2.3562 e^{-6}\, m^3\ \times 24\ h} = 20.09\ kg\ m^{-3}\ h^{-1}$$

$$\text{Productivity} = \frac{mmol\ of\ product}{reaction\ time \times g_{resin}} = \frac{5.59\ mmol}{24\ h\ \times 0.3\ g_{PS-Anth}} = 0.78\ mmol\ h^{-1}\ g_{resin}{}^{-1}$$

(*R*)-2-(3,4-dimethoxyphenethyl)-1,2,3,4-tetrahydroquinoline (3b)

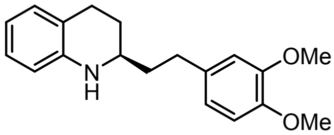

The general procedure was followed, collecting the reaction product for 12h in steady state and purified by column chromatography on silica gel (hexanes / EtOAc) to afford 0.719 g (84%) of the product as a white solid. The reported data match the literature.[10]

M. p. 57-58 °C (hexanes / EtOAc).

^{1}H NMR (300 MHz, $CDCl_3$) δ = 6.98 (t, *J* = 6.8 Hz, 2H), 6.84-6.75 (m, 3H), 6.62 (td, *J* = 7.4, 1.1 Hz, 1H), 6.47 (dd, *J* = 8.4, 1.1 Hz, 1H), 3.89 (d, *J* = 4.1, 6H), 3.78 (bs, 1H), 3.32 (dtd, *J* = 9.4, 6.3, 3.0 Hz, 1H), 2.89-2.68 (m, 4H), 2.01 (dddd, *J* = 13.0, 5.5, 4.5, 3.0 Hz, 1H), 1.83 (dtd, *J* = 8.8, 6.5, 0.9 Hz, 2H), 1.69 (dddd, *J* = 12.9, 10.2, 9.3, 5.8 Hz, 1H).

^{13}C-NMR {^{1}H} (75 MHz, $CDCl_3$) δ = 149.1, 147.4, 144.2, 134.5, 129.4, 126.9, 121.7, 120.3, 117.6, 114.6, 111.7, 111.4, 56.1, 56.0, 51.5, 38.4, 32.0, 28.0, 26.3.

HRMS (TOF+) m/z: Calcd. for $C_{19}H_{24}NO_2$ $[M+H]^+$: 298.1802; Found: 298.1805.

HPLC (chiral): Chiralpak-AD-H, n-heptane/i-PrOH 95:5, 1.2 mL/min, 40 °C, detection at 254 nm.

Residence time: 12.6 min (major), 14.0 (minor). 74:26 er.

$$\text{TON} = \frac{mmoles\ limiting\ reactant}{mmoles\ catalyst} \times yield = \frac{2.88}{0.021} \times 0.84 = 115.2$$

$$\text{STY} = \frac{mass\ of\ product}{volume\ of\ reactor\ \times reaction\ time} = \frac{0.719\ e^{-3} kg}{2.3562 e^{-6}\ m^3\ \times 12\ h} = 25.43\ kg\ m^{-3}\ h^{-1}$$

$$\text{Productivity} = \frac{mmol\ of\ product}{reaction\ time \times g_{resin}} = \frac{2.42\ mmol}{12\ h\ \times 0.3\ g_{PS-Anth}} = 0.67\ mmol\ h^{-1}\ g_{resin}{}^{-1}$$

(*R*)-2-(2-(benzo[d][1,3]dioxol-5-yl)ethyl)-1,2,3,4-tetrahydroquinoline (3c)

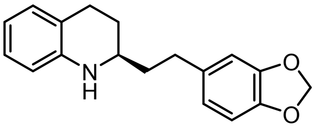

The general procedure was followed, collecting the reaction product for 12h in steady state and purified by column chromatography on silica gel (hexanes / EtOAc) to afford 0.713 g (88%) of the product as a white solid. The reported data match the literature.[11]

M. p. 64-65 °C (hexanes / EtOAc).

^{1}H NMR (300 MHz, $CDCl_3$) δ = 7.00 (t, *J* = 7.2 Hz, 2H), 6.79-6.61 (m, 4H), 6.49 (dd, *J* = 8.4, 1.0 Hz, 1H), 5.95 (s, 2H), 3.79 (bs, 1H), 3.31 (dtd, *J* = 9.4, 6.4, 3.0 Hz, 1H), 2.90-2.66 (m, 4H), 2.01 (dddd, *J* = 12.9, 5.5, 4.4, 3.0 Hz, 1H), 1.85-1.75 (m, 2H), 1.73-1.63 (m, 1H).

^{13}C {^{1}H} NMR (75 MHz, $CDCl_3$) δ = 147.8, 145.8, 144.6, 135.7, 129.4, 126.8, 121.4, 121.1, 117.1, 114.2, 108.9, 108.3, 100.9, 51.1, 38.6, 32.0, 28.0, 26.3.

HRMS (TOF+), m/z: Calcd. for for $C_{18}H_{20}NO_2$ $[M+H]^+$: 282.1489; Found: 282.1488.

HPLC (chiral): Chiralpak-AD-H, n-heptane/i-PrOH 99:1, 1.2 mL/min, 40 °C, detection at 254 nm. Residence time: 21.6 min (major), 28.2 (minor). 90:10 er.

$$\text{TON} = \frac{mmoles\ limiting\ reactant}{mmoles\ catalyst} \times yield = \frac{2.88}{0.021} \times 0.88 = 120.7$$

$$\text{STY} = \frac{mass\ of\ product}{volume\ of\ reactor\ \times reaction\ time} = \frac{0.713\ e^{-3} kg}{2.3562 e^{-6}\ m^3\ \times 12\ h} = 25.22\ kg\ m^{-3}\ h^{-1}$$

$$\text{Productivity} = \frac{mmol\ of\ product}{reaction\ time \times g_{resin}} = \frac{2.53\ mmol}{12\ h\ \times 0.3\ g_{PS-Anth}} = 0.70\ mmol\ h^{-1}\ g_{resin}{}^{-1}$$

Chiral HPLC chromatograms of **3**

(*R*)-2-pentyl-1,2,3,4-tetrahydroquinoline (3a)

<Chromatogram>

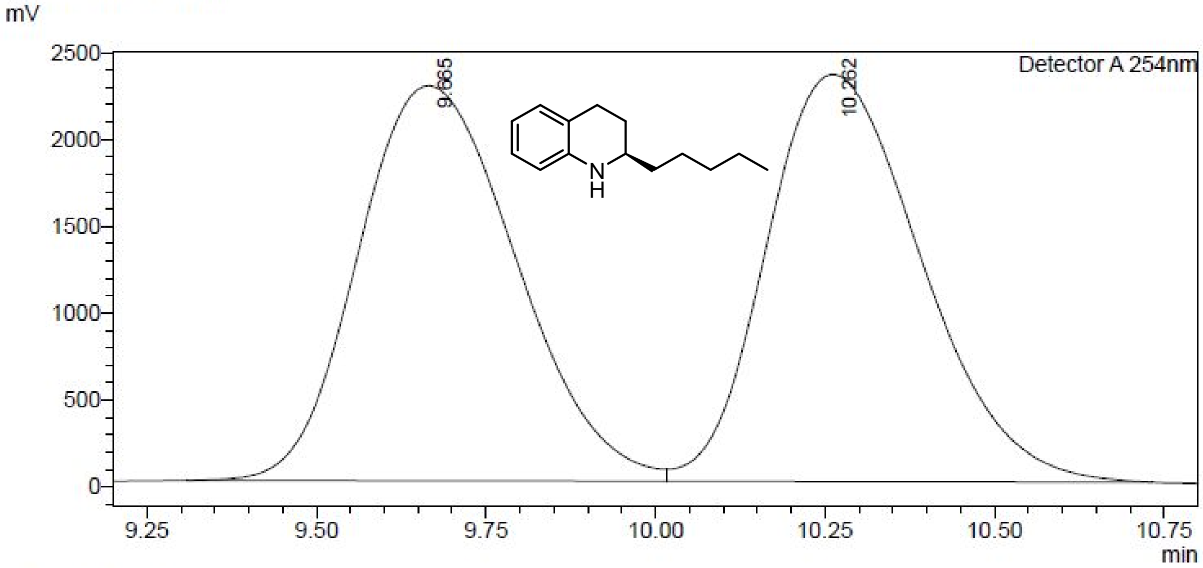

<Peak Table>

Detector A 254nm

Peak#	Ret. Time	Area	Height	Area%
1	9.665	36894020	2274901	49.802
2	10.262	37187214	2343025	50.198
Total		74081233	4617926	100.000

<Chromatogram>

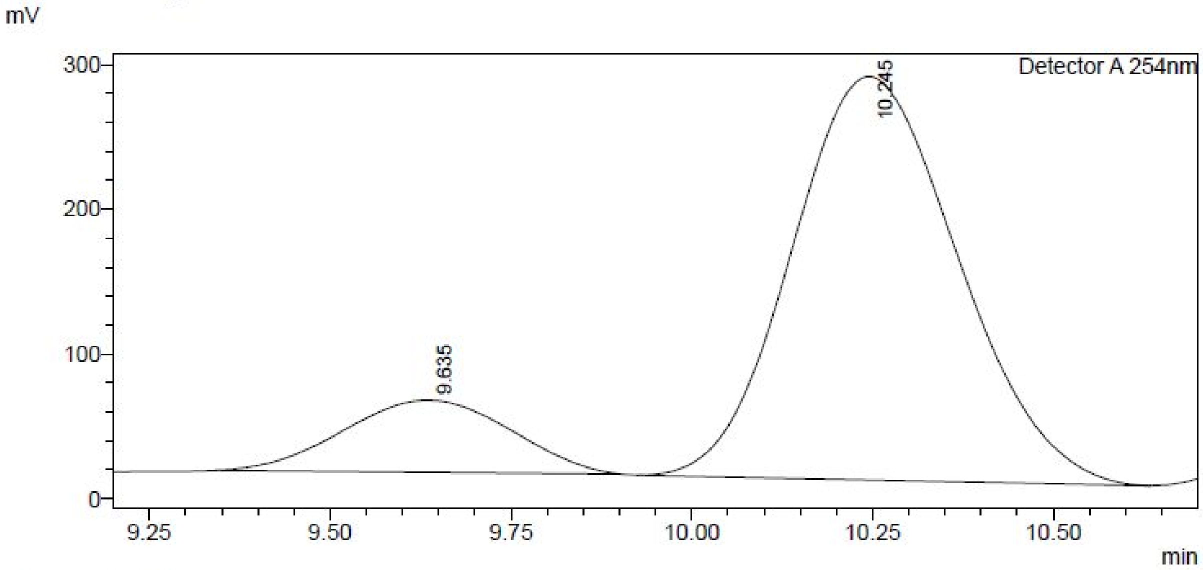

<Peak Table>

Detector A 254nm

Peak#	Ret. Time	Area	Height	Area%
1	9.635	795621	49699	15.183
2	10.245	4444724	278599	84.817
Total		5240345	328298	100.000

(*R*)-2-(3,4-dimethoxyphenethyl)-1,2,3,4-tetrahydroquinoline (3b)

<Chromatogram>

mV

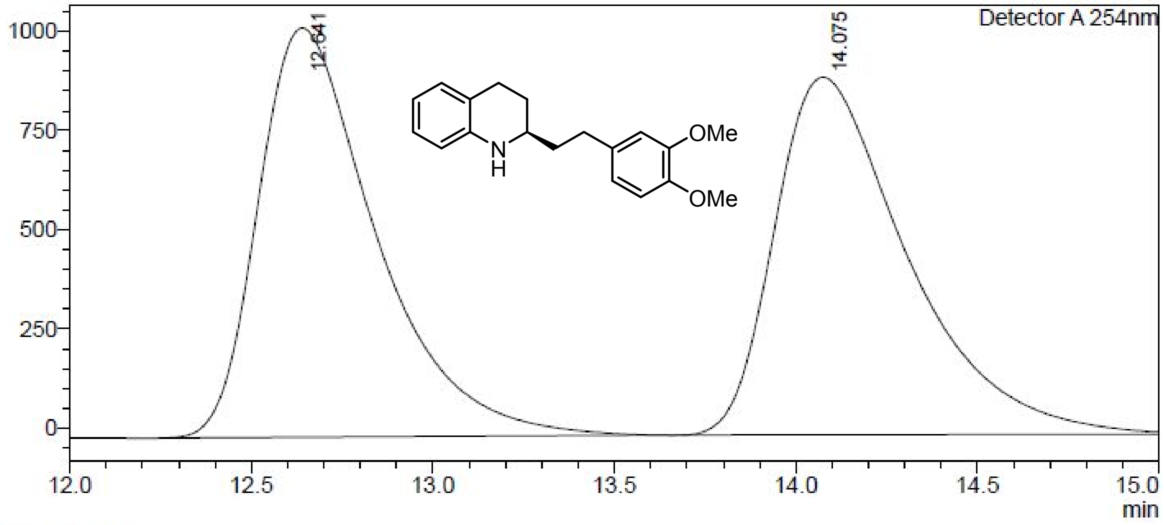

<Peak Table>

Detector A 254nm

Peak#	Ret. Time	Area	Height	Area%
1	12.641	23312335	1032411	50.303
2	14.075	23031504	902463	49.697
Total		46343839	1934874	100.000

<Chromatogram>

mV

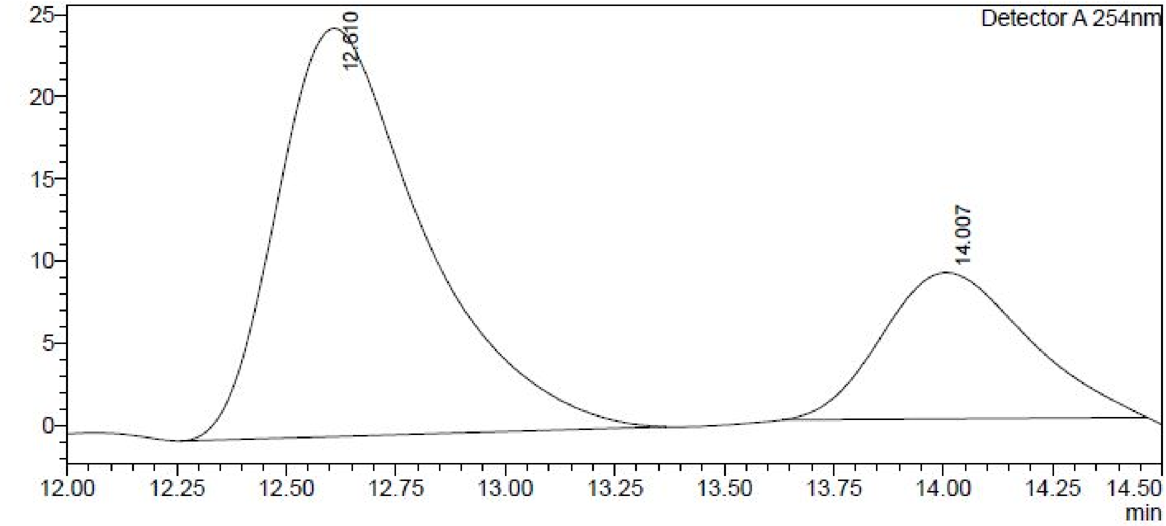

<Peak Table>

Detector A 254nm

Peak#	Ret. Time	Area	Height	Area%
1	12.610	577272	24812	73.702
2	14.007	205981	8893	26.298
Total		783253	33705	100.000

(*R*)-2-(2-(benzo[d][1,3]dioxol-5-yl)ethyl)-1,2,3,4-tetrahydroquinoline (3c)

<Chromatogram>

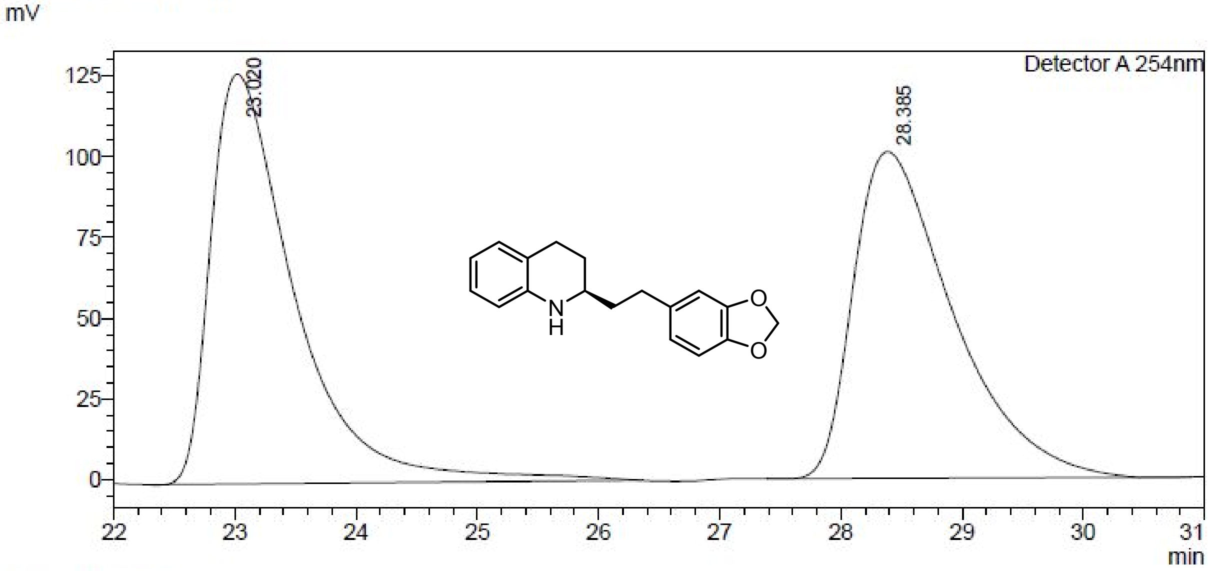

<Peak Table>

Detector A 254nm

Peak#	Ret. Time	Area	Height	Area%
1	23.020	6124518	126930	51.452
2	28.385	5778766	101122	48.548
Total		11903284	228052	100.000

<Chromatogram>

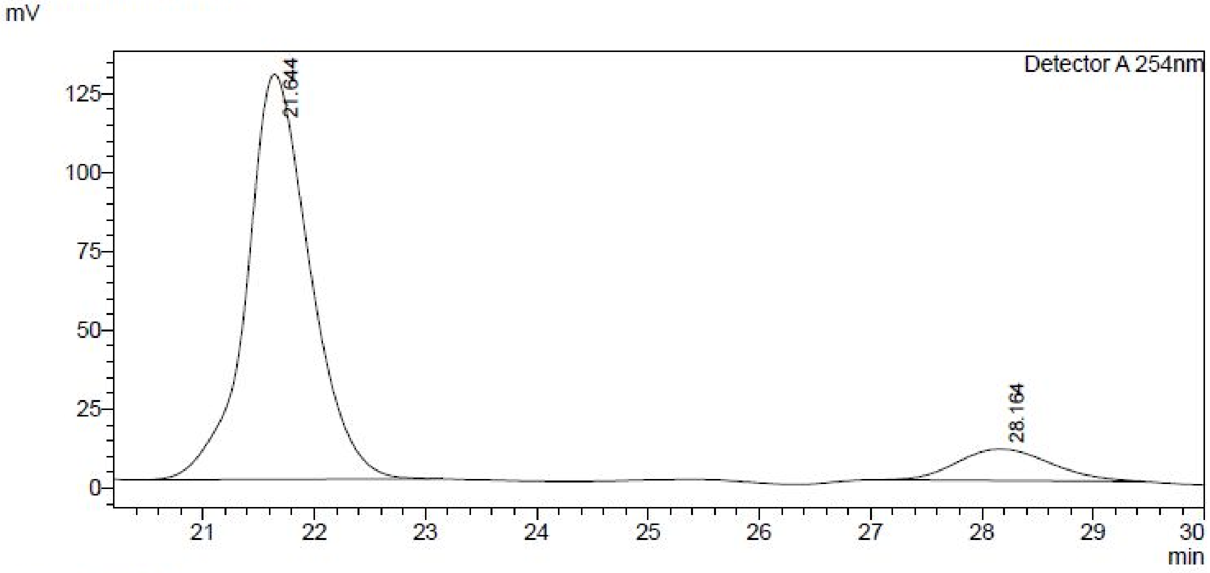

<Peak Table>

Detector A 254nm

Peak#	Ret. Time	Area	Height	Area%
1	21.644	5096617	128466	89.701
2	28.164	585187	9999	10.299
Total		5681804	138465	100.000

NMR spectra of BINOL derivatives **B-F**

(*R*)-6,6'-dibromo-3,3'-diiodo-[1,1'-binaphthalene]-2,2'-diol (BINOL-B) ($CDCl_3$, ^1H 300 MHz, ^{13}C 75 MHz)

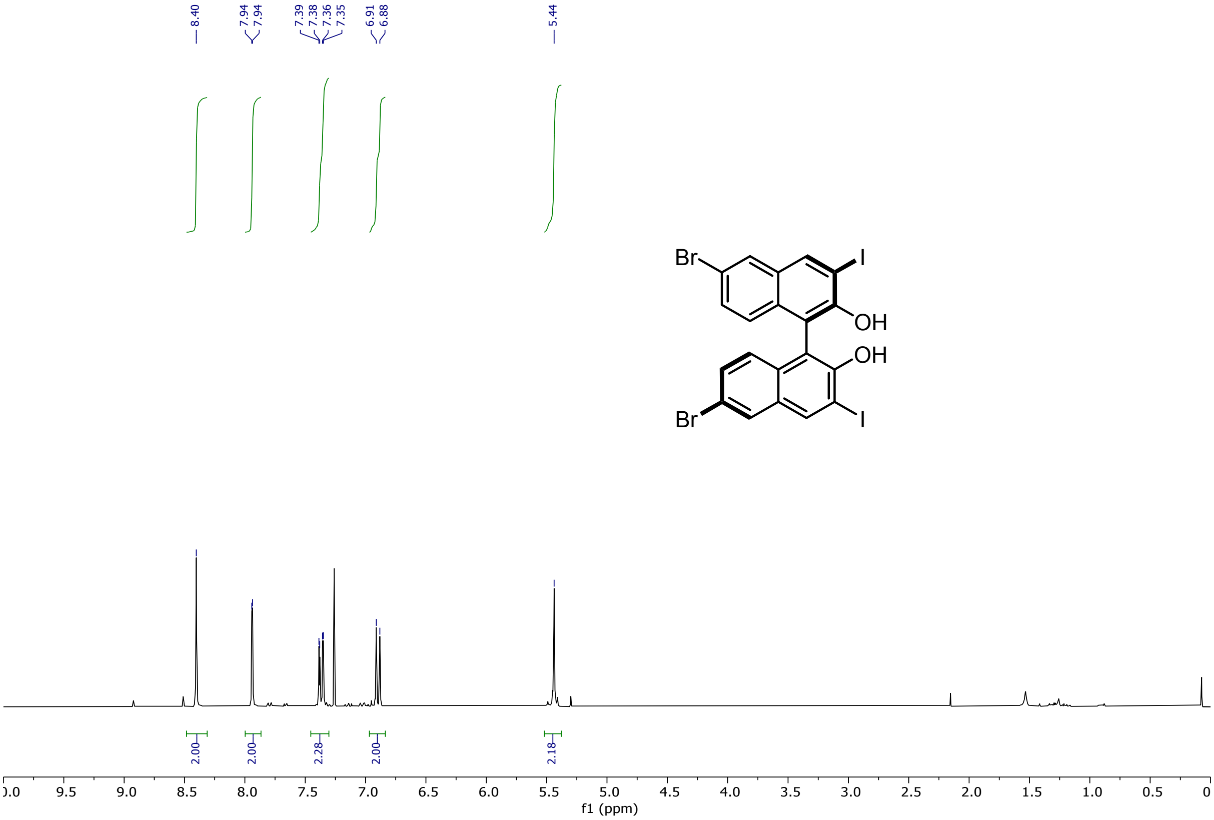

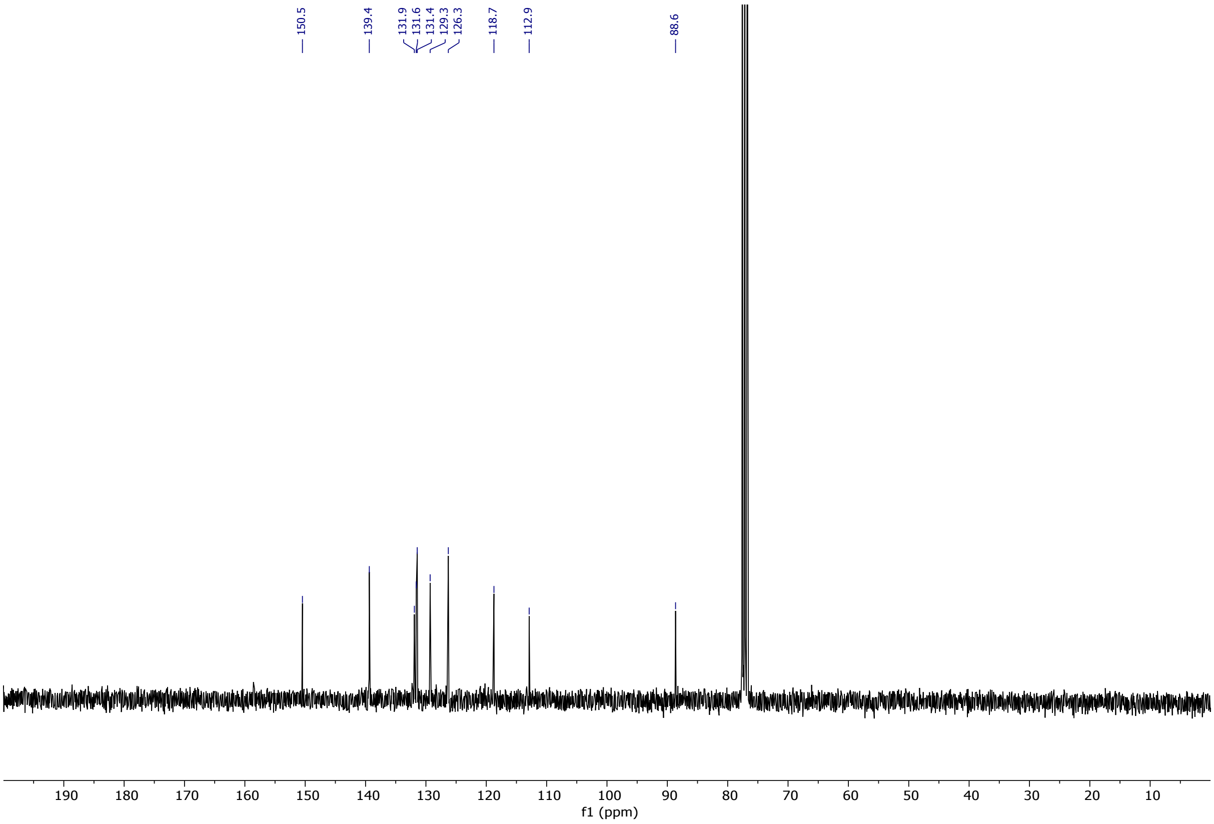

(*R*)-6,6'-dibromo-3,3'-diiodo-2,2'-bis(methoxymethoxy)-1,1'-binaphthalene (BINOL-C) ($CDCl_3$, ^1H 300 MHz, ^{13}C 75 MHz)

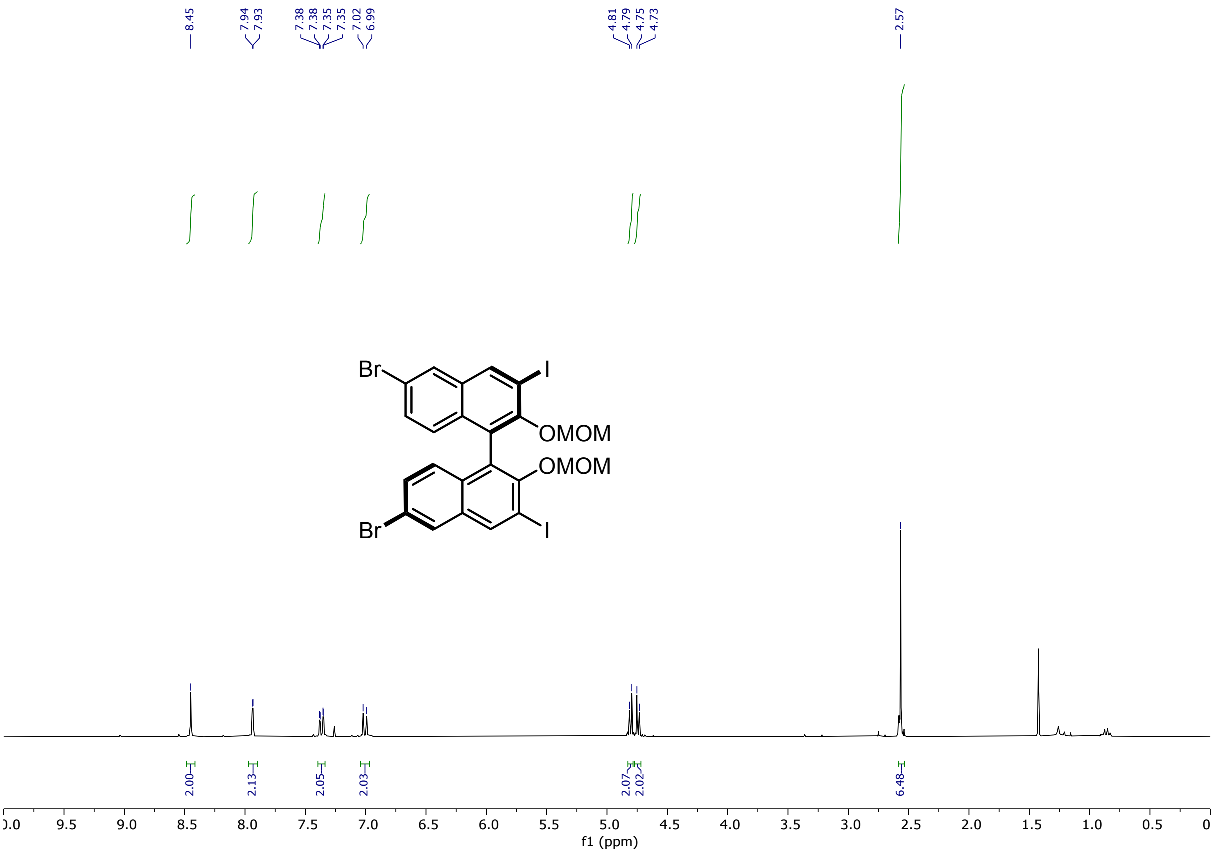

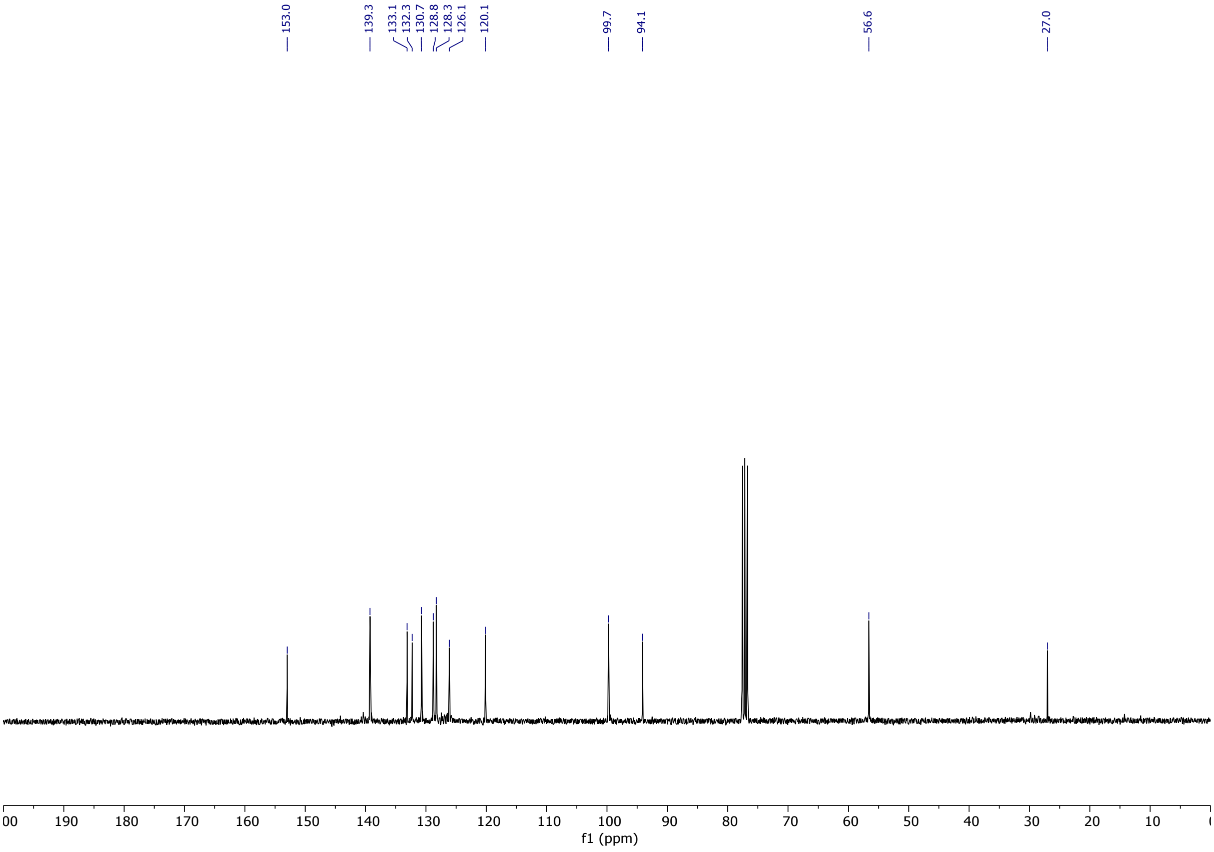

(*R*)- 6,6'-dibromo-3,3'-di(anthracen -9-yl)-[1,1'-binaphthalene]-2,2'-diol (BINOL-D) ($CDCl_3$, ^1H 300 MHz, ^{13}C 75 MHz)

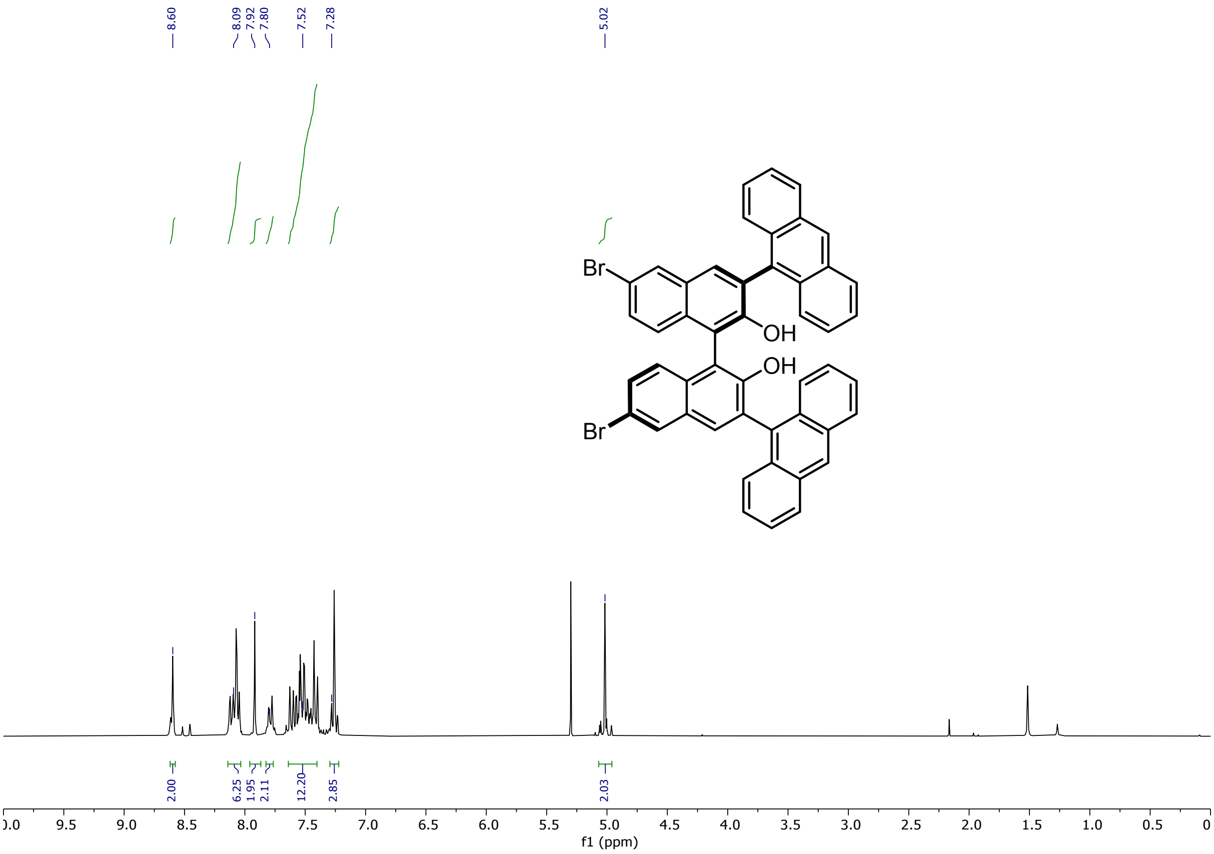

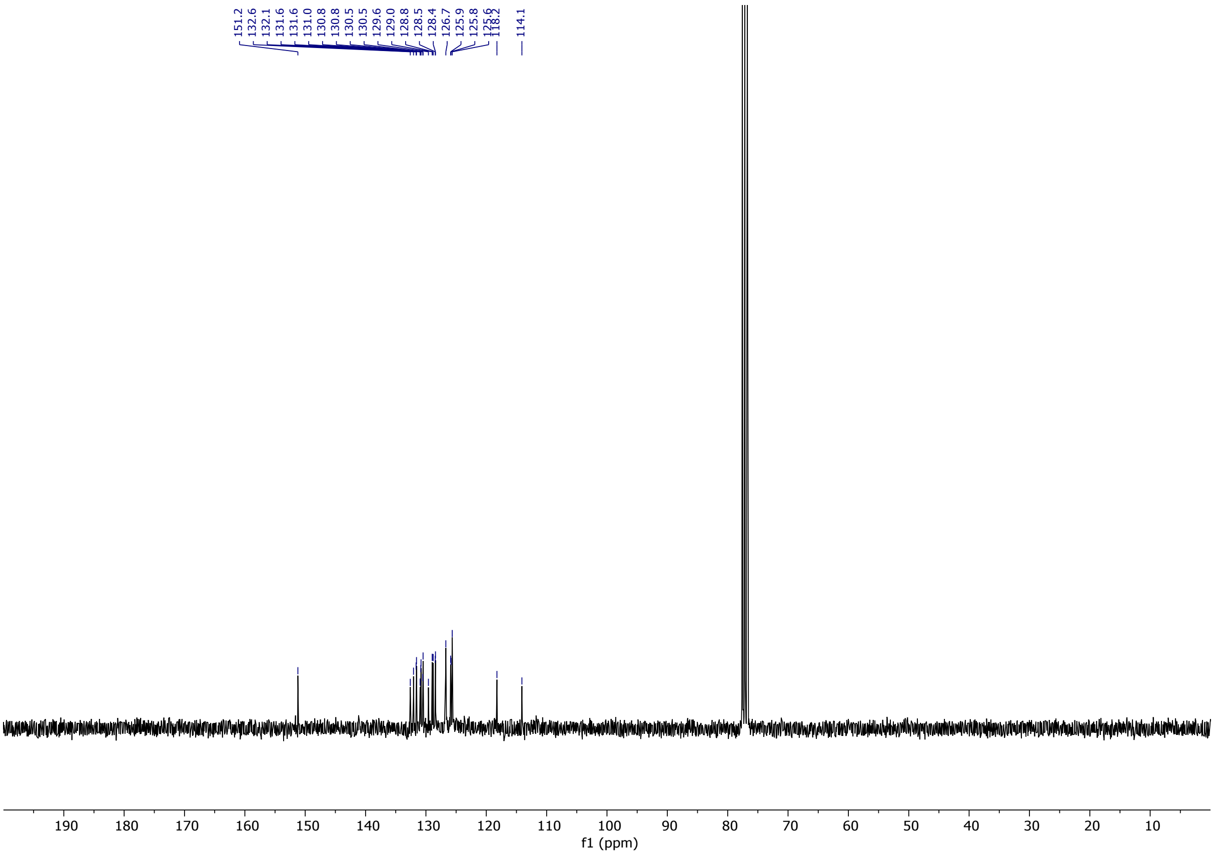

(*R*)-3,3'-di(anthracen-9-yl)-6,6'-bis(4-vinylphenyl)-[1,1'-binaphthalene]-2,2'-diol (BINOL-E) ($CDCl_3$, ^1H 300 MHz, ^{13}C 75 MHz)

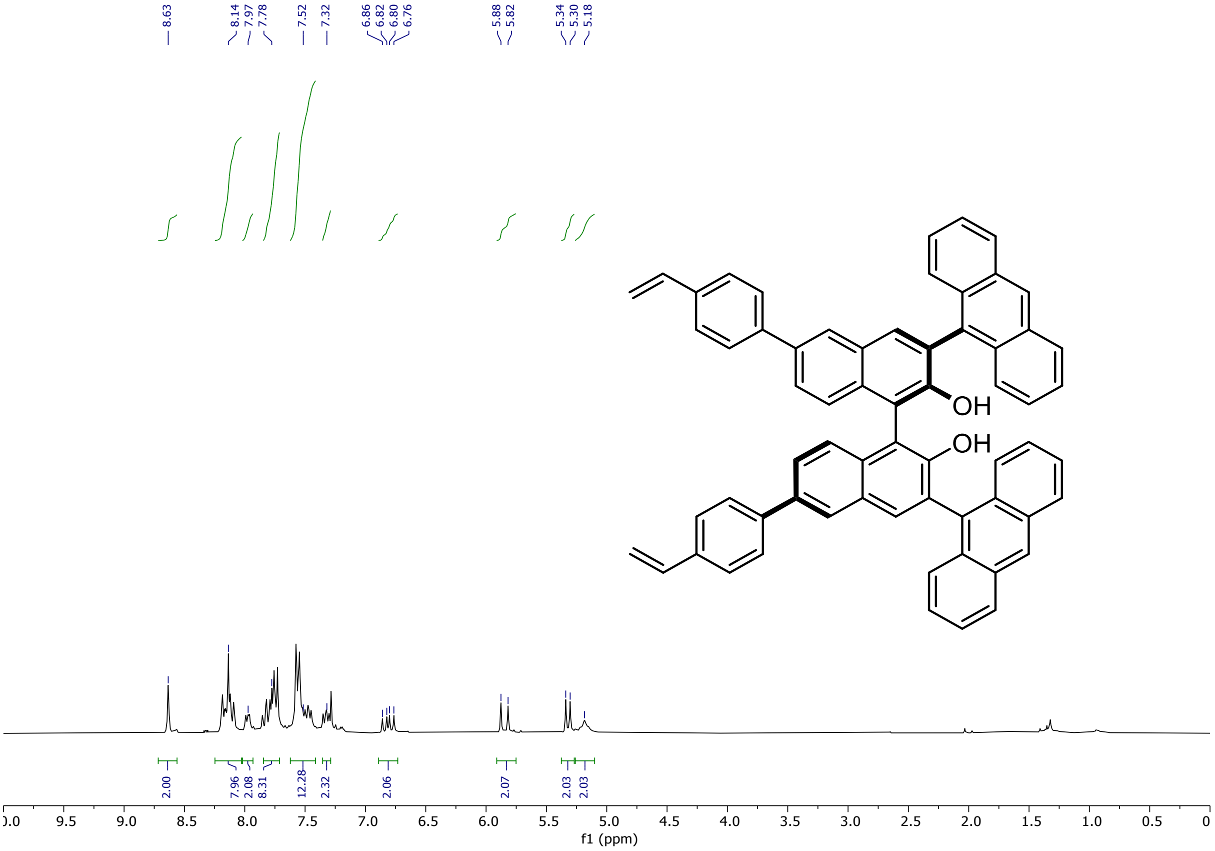

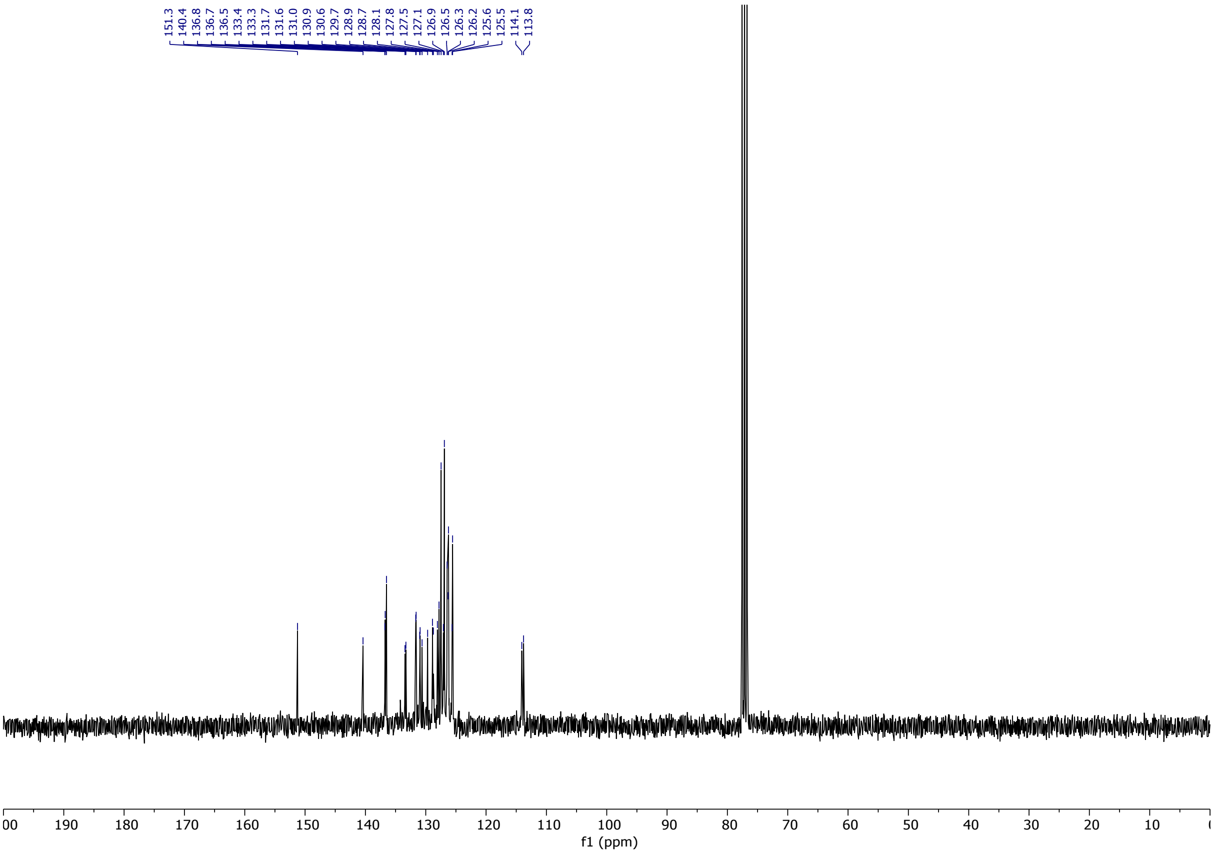

(*R*)-2,6-di(anthracen-9-yl)-4-hydroxy-9,14-bis(4-vinylphenyl)dinaphtho[2,1-d:1',2'-f][1,3,2]dioxaphosphepine 4-oxide (BINOL-F) (DMSO, ^{1}H 300 MHz, ^{13}C 75 MHz, ^{31}P 121 MHz)

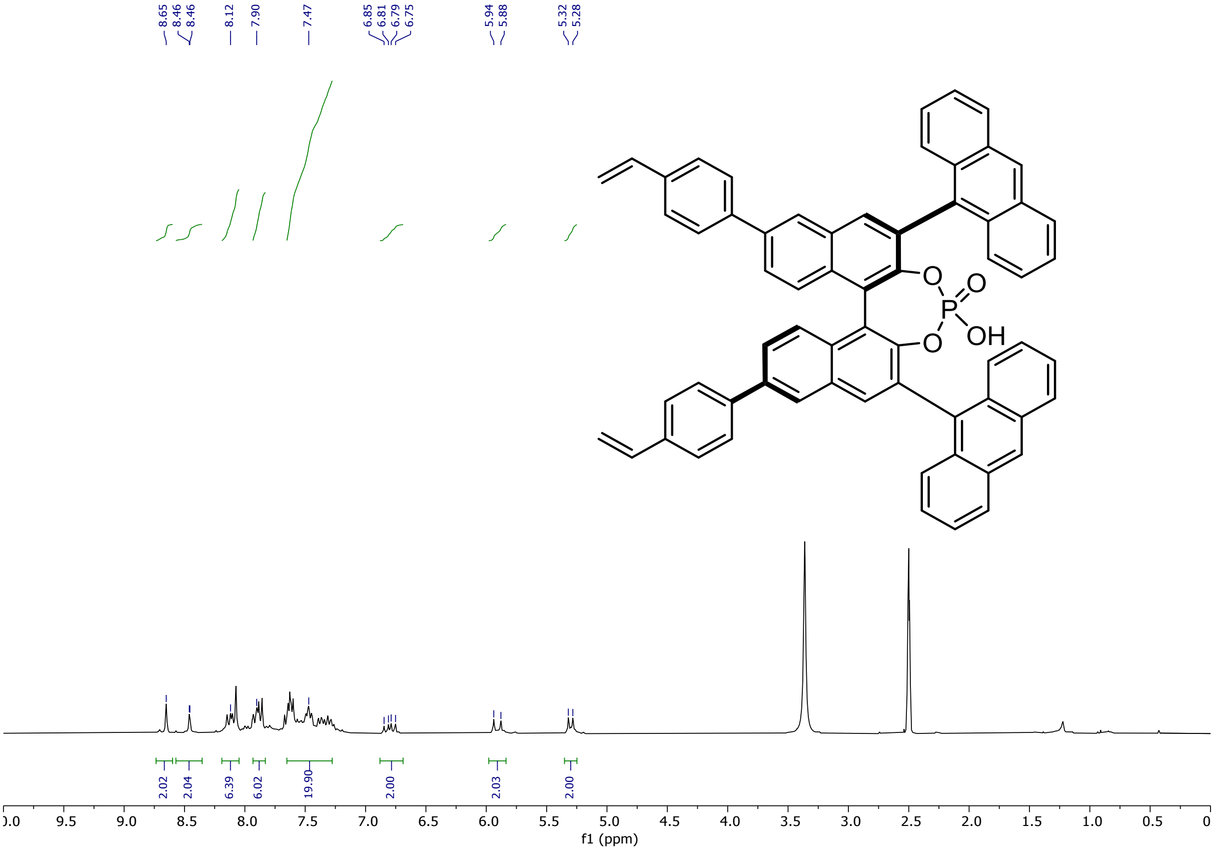

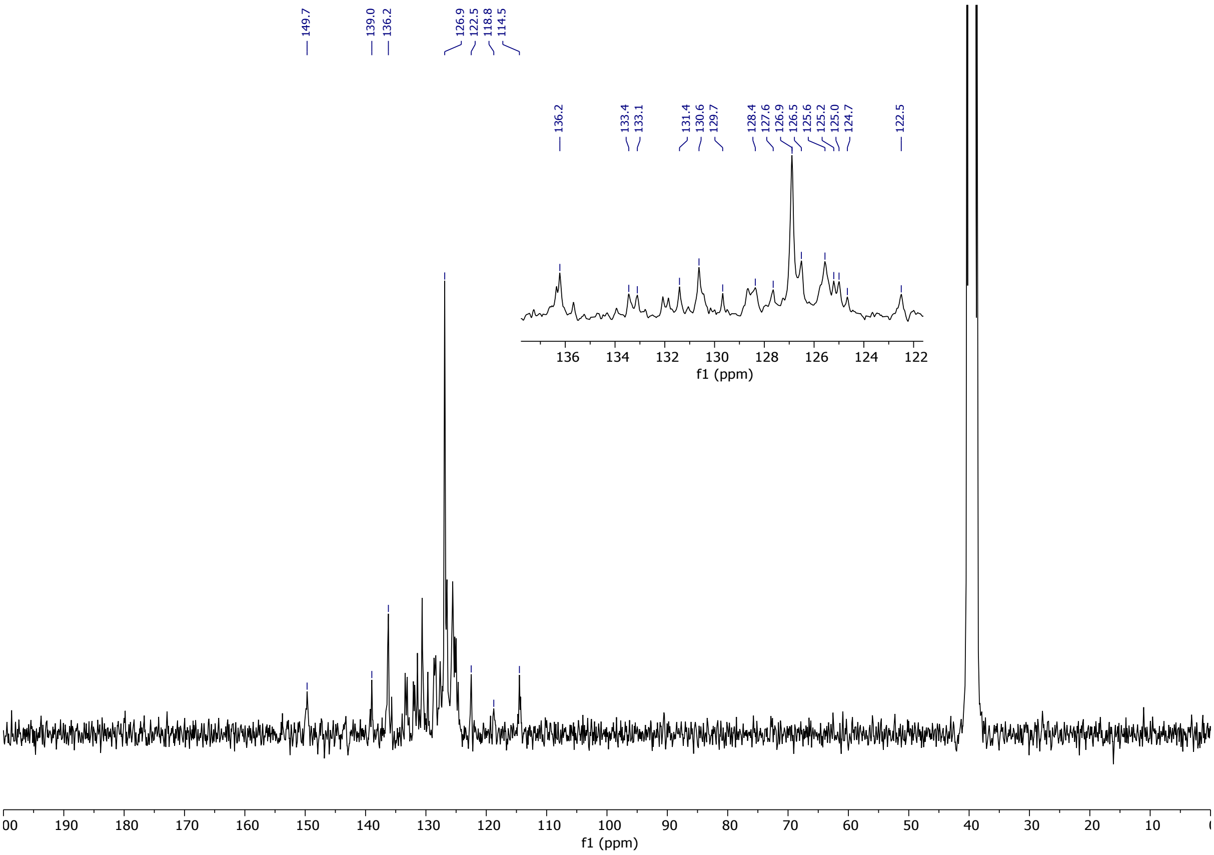

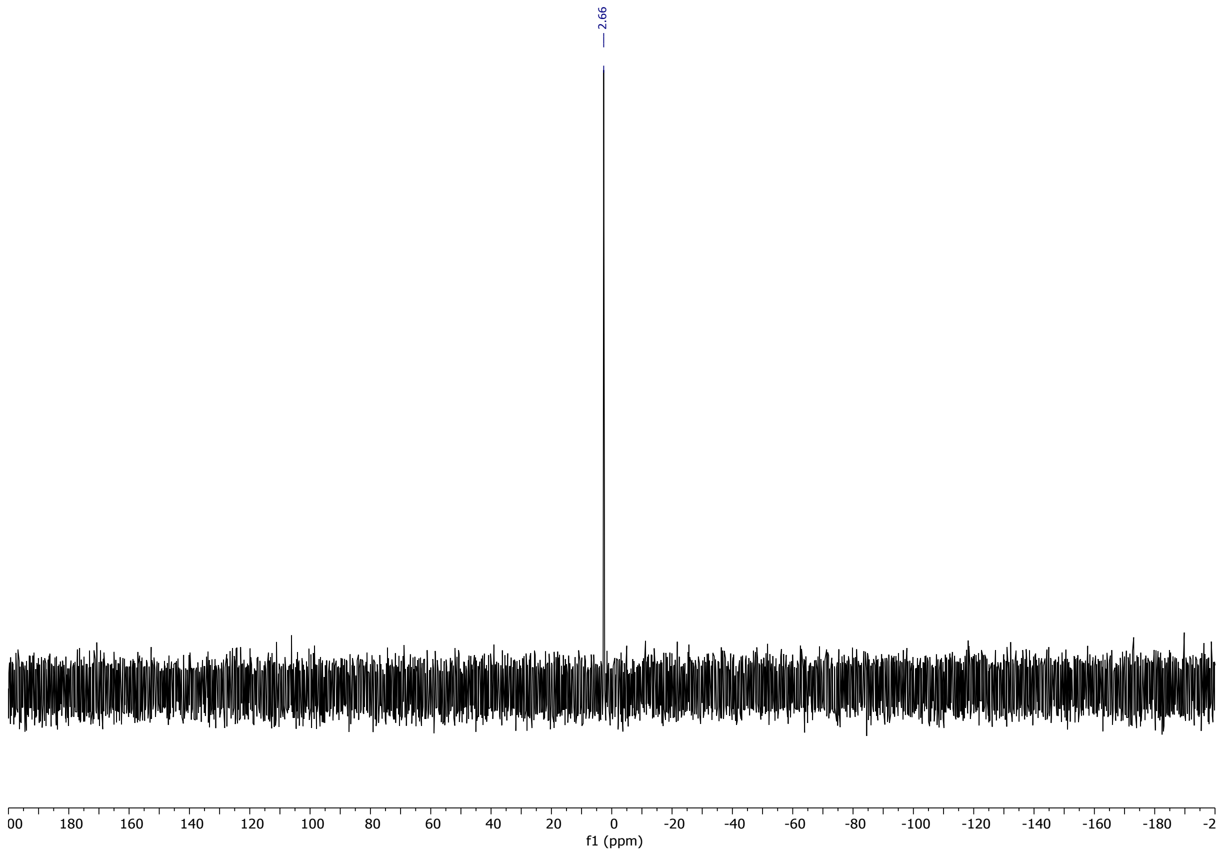
2.66
00
180
160
140
120
100
80
60
40
20
0
-20
-40
-60
-80
-100
-120
-140
-160
-180
-2
f1 (ppm)

NMR spectra of **3**

(*R*)-2-pentyl-1,2,3,4-tetrahydroquinoline (3a) ($CDCl_3$, ^1H 300 MHz, ^{13}C 75 MHz)

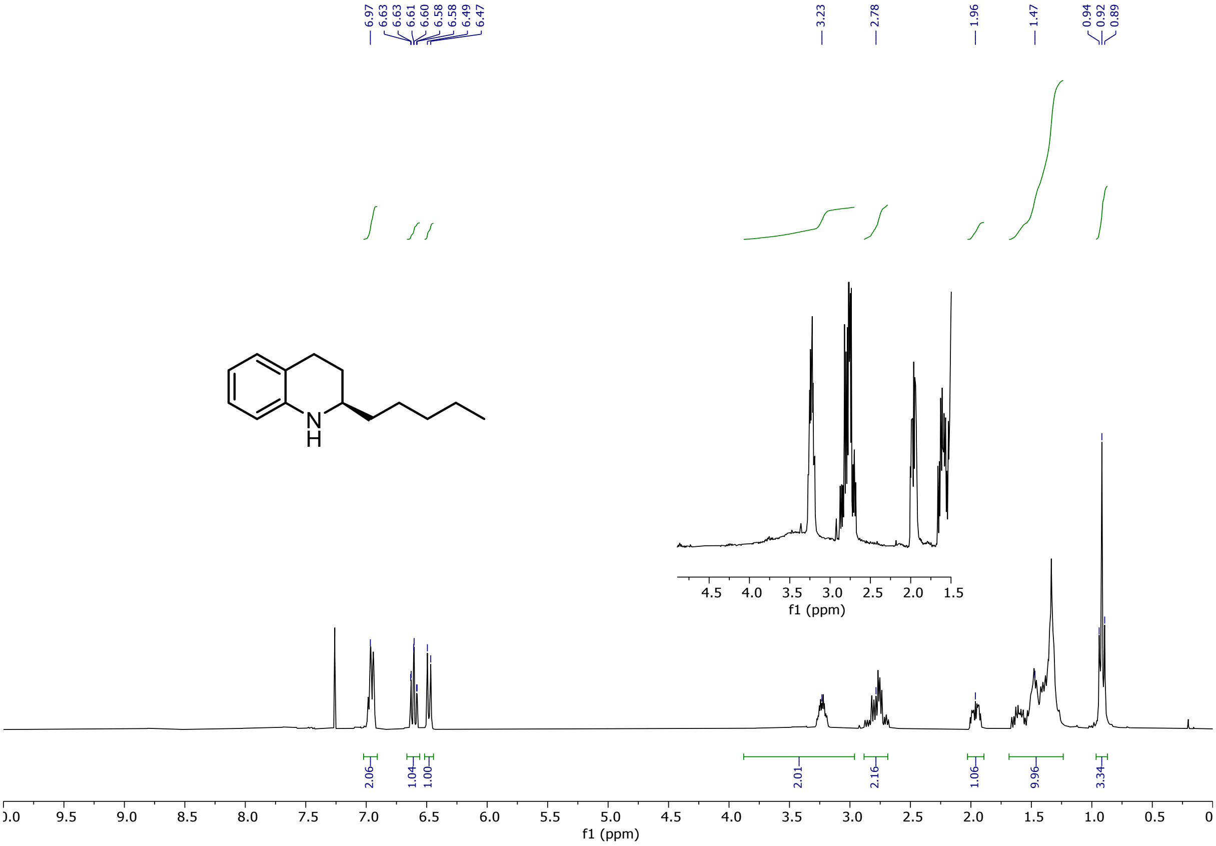

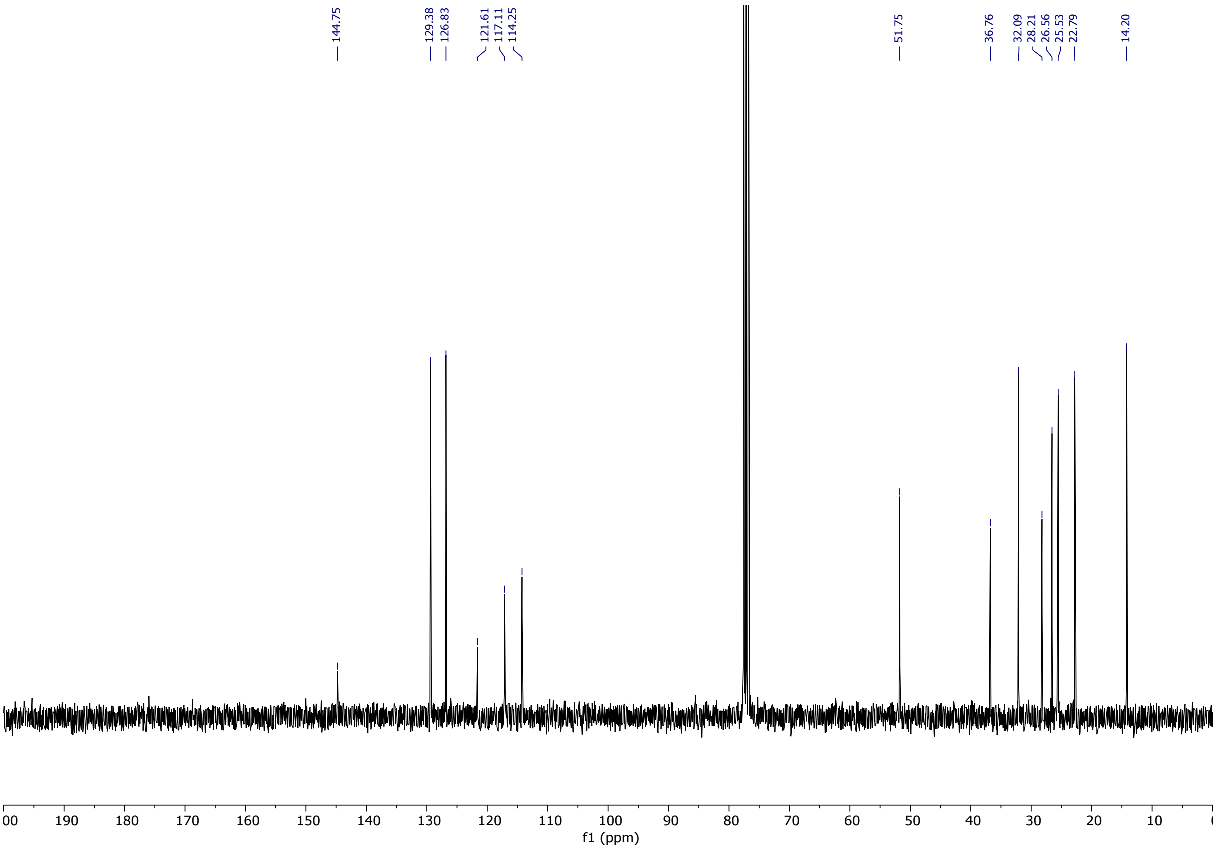

(*R*)-2-(3,4-dimethoxyphenethyl)-1,2,3,4-tetrahydroquinoline (3b) ($CDCl_3$, ^{1}H 300 MHz, ^{13}C 75 MHz)

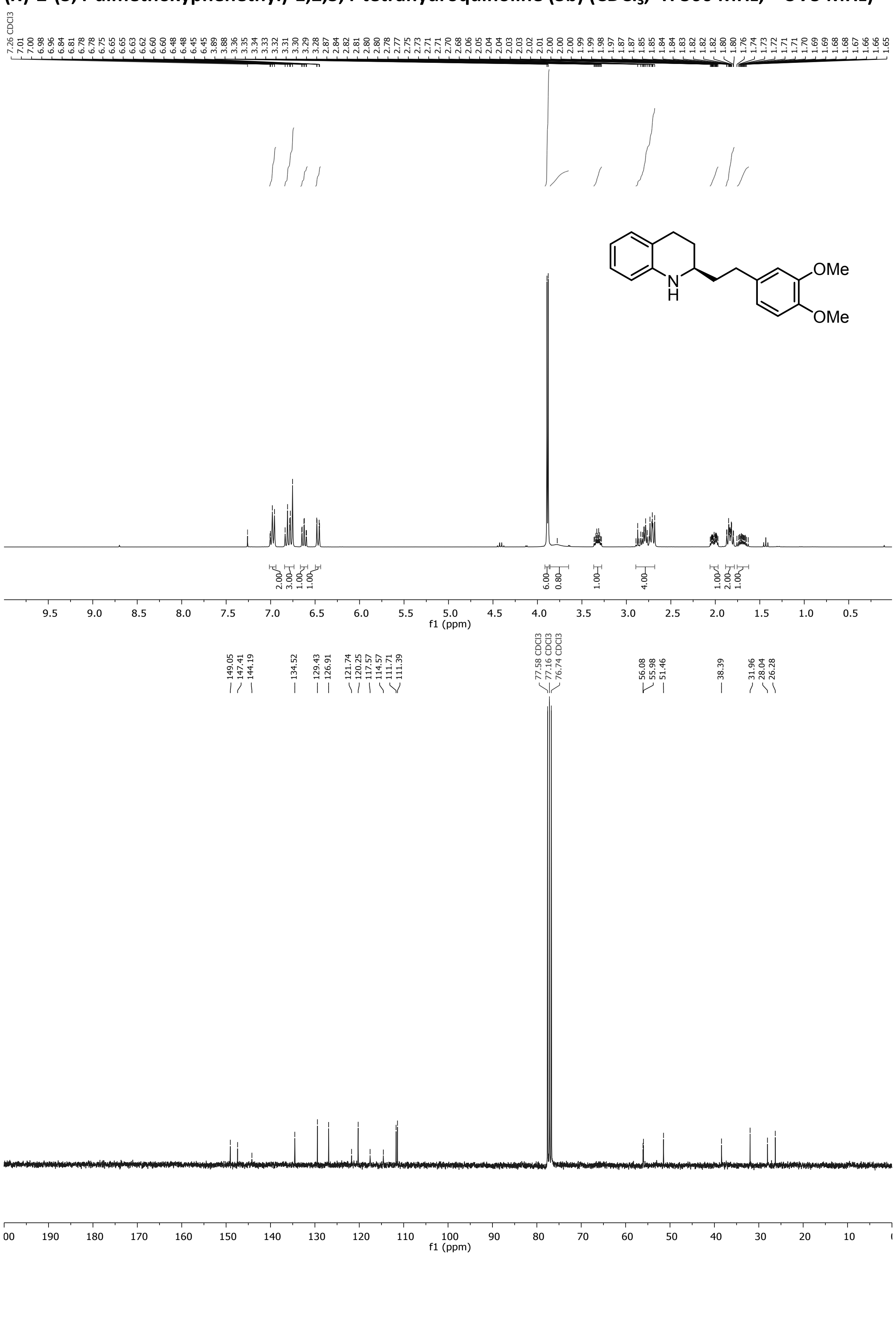

(*R*)-2-(2-(benzo[d][1,3]dioxol-5-yl)ethyl)-1,2,3,4-tetrahydroquinoline (3c) ($CDCl_3$, ^1H 300 MHz, ^{13}C 75 MHz)

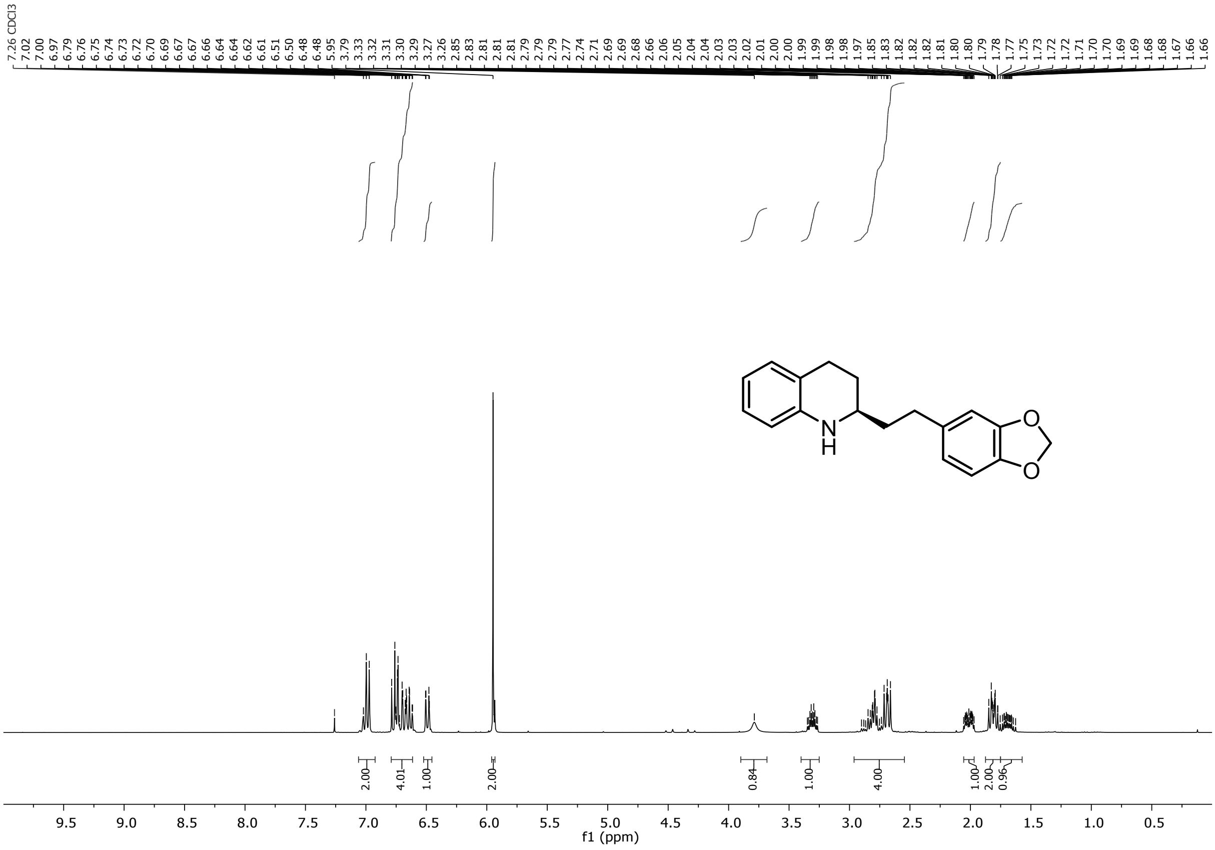

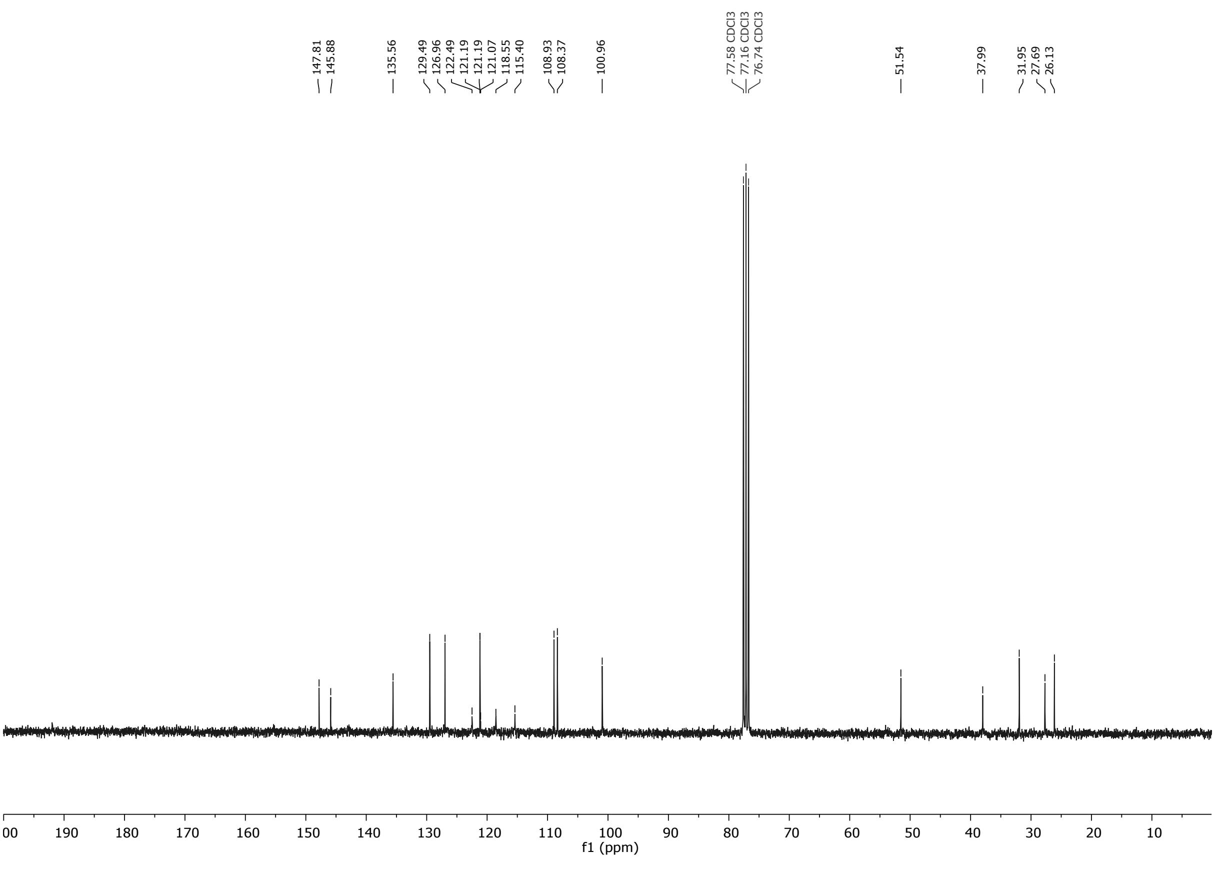

References

S1 F. Mao, J. Yan, J. Li, X. Jia, H. Miao, Y. Sun, L. Huang and X. Li, New multi-target-directed small molecules against Alzheimer's disease: A combination of resveratrol and clioquinol, *Org. Biomol. Chem.*, 2014, **12**, 5936–5944.

S2 S. Fu, L. Wang, H. Dong, J. Yu, L. Xu and J. Xiao, Facile synthesis of 2-alkenylazaarenes via dehydrative coupling of 2-methylazaarenes with aldehydes 'on water', *Tetrahedron Lett.*, 2016, **57**, 4533–4536.

S3 M. Lautens and M. Yoshida, Rhodium-catalyzed addition of arylboronic acids to alkynyl aza-heteroaromatic compounds in water, *J. Org. Chem.*, 2003, **68**, 762–769.

S4 S. Paul, M. Bhakat and J. Guin, Radical C−H Acylation of Nitrogen Heterocycles Induced by an Aerobic Oxidation of Aldehydes, *Chem. Asian J.*, 2019, **14**, 3154–3160.

S5 Z. W. Li, T. L. Wang, Y. M. He, Z. J. Wang, Q. H. Fan, J. Pan and L. J. Xu, Air-stable and phosphine-free iridium catalysts for highly enantioselective hydrogenation of quinoline derivatives, *Org. Lett.*, 2008, **10**, 5265–5268.

S6 A. Maestro, S. B. Ötvös, G. Auer and C. O. Kappe, General and versatile synthesis of highly recyclable chiral phosphoric acid organocatalysts, *Org. Chem. Front.*, 2024, Advance Article.

S7 Y. Yamashita, H. Ishitani, H. Shimizu and S. Kobayashi, Highly anti-selective asymmetric aldol reactions using chiral zirconium catalysts. Improvement of activities, structure of the novel zirconium complexes, and effect of a small amount of water for the preparation of the catalysts, *J. Am. Chem. Soc.*, 2002, **124**, 3292–3302.

S8 C. R. McElroy, A. Constantinou, L. C. Jones, L. Summerton and J. H. Clark, Towards a holistic approach to metrics for the 21st century pharmaceutical industry, *Green Chem.*, 2015, **17**, 3111–3121.

S9 S. B. Ötvös, M. A. Pericàs and C. O. Kappe, Multigram-scale flow synthesis of the chiral key intermediate of (-)-paroxetine enabled by solvent-free heterogeneous organocatalysis, *Chem. Sci.*, 2019, **10**, 11141–11146.

S10 C. Liu, M. Wang, S. Liu, Y. Wang, Y. Peng, Y. Lan and Q. Liu, Manganese-Catalyzed Asymmetric Hydrogenation of Quinolines Enabled by π–π Interaction**, *Angew. Chem. Int. Ed.*, 2021, **60**, 5108–5113.

S11 J. Das, M. Vellakkaran, M. Sk and D. Banerjee, Iron-Catalyzed Coupling of Methyl N-Heteroarenes with Primary Alcohols: Direct Access to E-Selective Olefins, *Org. Lett.*, 2019, **21**, 7514–7518.
